# Molecular heterogeneity in major urinary proteins of *Mus musculus* subspecies: potential candidates involved in speciation

**DOI:** 10.1038/srep44992

**Published:** 2017-03-24

**Authors:** Jane L. Hurst, Robert J. Beynon, Stuart D. Armstrong, Amanda J. Davidson, Sarah A. Roberts, Guadalupe Gómez-Baena, Carole M. Smadja, Guila Ganem

**Affiliations:** 1 Mammalian Behaviour and Evolution Group, Institute of Integrative Biology, University of Liverpool, Leahurst Campus, Neston, CH64 7TE, UK; 2 Centre for Proteome Research, Institute of Integrative Biology, University of Liverpool, Liverpool, L69 7ZB, UK; 3 Institut des Sciences de l’Evolution de Montpellier UMR5554 (UM, CNRS, IRD, EPHE), Université de Montpellier, CC 065, 34095 Montpellier Cedex 05, France

## Abstract

When hybridisation carries a cost, natural selection is predicted to favour evolution of traits that allow assortative mating (reinforcement). Incipient speciation between the two European house mouse subspecies, *Mus musculus domesticus* and *M.m.musculus*, sharing a hybrid zone, provides an opportunity to understand evolution of assortative mating at a molecular level. Mouse urine odours allow subspecific mate discrimination, with assortative preferences evident in the hybrid zone but not in allopatry. Here we assess the potential of MUPs (major urinary proteins) as candidates for signal divergence by comparing MUP expression in urine samples from the Danish hybrid zone border (contact) and from allopatric populations. Mass spectrometric characterisation identified novel MUPs in both subspecies involving mostly new combinations of amino acid changes previously observed in *M.m.domesticus*. The subspecies expressed distinct MUP signatures, with most MUPs expressed by only one subspecies. Expression of at least eight MUPs showed significant subspecies divergence both in allopatry and contact zone. Another seven MUPs showed divergence in expression between the subspecies only in the contact zone, consistent with divergence by reinforcement. These proteins are candidates for the semiochemical barrier to hybridisation, providing an opportunity to characterise the nature and evolution of a putative species recognition signal.

Sexual signals are sometimes the only phenotypic traits distinct between species, and divergence of such traits could be important isolating mechanisms^1^,^2^,^3^. Chemosignals (molecules involved in odour based communication) are involved in speciation in a large diversity of taxa^4^. Very few of the molecules involved have yet been identified and these have mostly concerned insect species e.g. refs 5, 6, 7. Despite growing knowledge of the molecular identity of odours involved in social and sexual communication in mammals^8^,^9^,^10^, molecules involved in odour-based species recognition remain elusive. The present study addresses this question in the framework of incipient speciation between the two European subspecies of the house mouse, *Mus musculus domesticus* and *M. m. musculus* (hereafter *domesticus* and *musculus*)^11^,^12^,^13^.

After evolution in allopatry from the sub-Indian continent, the house mouse colonised Europe as two subspecies that occupy different parts of the continent^14^,^15^. Whenever the two subspecies come into contact they hybridize. The hybrid zone is relatively narrow (typically 40 km across) and stretches from Norway to the Black Sea (Fig. 1). Hybrids derived from the two subspecies have reduced fitness compared to non-hybrids^16^,^17^,^18^, and theory predicts the evolution of assortative mating in the hybrid zone as a response to this selection against hybridisation (a process called reinforcement^19^,^20^). Several studies, involving classical laboratory two-way choice tests, have consistently pointed out assortative mate preference in both wild derived and wild trapped populations from the border of the hybrid zone of the two subspecies^12^,^21^,^22^,^23^,^24^,^25^,^26^,^27^, but not in their allopatric counterparts^12^,^13^,^22^,^23^,^24^,^26^,^27^. This is a pattern expected under a reinforcement scenario. Olfactory mating signals present in mouse urine were pointed out as potential candidates for this divergence^22^,^25^. Hence we targeted a well-known family of semiochemicals (chemicals used for communication between organisms) for which roles in social and sexual communication have been established: the major urinary proteins, known as MUPs^28^,^29^,^30^,^31^,^32^,^33^,^34^,^35^,^36^,^37^,^38^. Comparison of the two European subspecies of the house mouse from the border of the Bavarian-Czech Republic hybrid zone has revealed quantitative variation in the expression of mRNA for MUPs in the liver and in total urinary MUP concentration^39^. These results suggested that MUPs are expressed in male urine at a higher level in *musculus* than in *domesticus*, while levels in female urine were similar between the subspecies. The present study aims to characterise divergence between the two subspecies in odours that underpin mate preference and identify semiochemicals that would be likely candidates for bioassay of subspecies scent preference. This study focuses on the involatile urinary MUPs, although the odour bouquet involved in subspecies assortative preference most probably comprises several different components, including volatile molecules^40^ and may work in concert with non-urinary components such as salivary androgen-binding proteins^41^.

MUPs are a family of 18–19 kDa proteins that are encoded by a multigene cluster on mouse chromosome IV^42^,^43^. They have predominantly been studied in *domesticus* and in laboratory mice, which are subspecies hybrids but carry *domesticus*-derived genes in this region of the chromosome^44^. In the C57BL/6J laboratory mouse (which provides the reference genome), there are at least 21 functional *Mup* genes, not all of which are expressed in urine. In the central region of the *Mup* cluster, the gene products share very high sequence similarity of over 97%, with five genes in the reference genome encoding identical mature proteins. MUPs encoded in the peripheral regions are more diverse^45^. Although MUPs are expressed in different tissues, the predominant route of expression for most MUPs is synthesis in the liver, followed by passage through the glomerular filter and excretion in urine. The MUPs are eight stranded beta barrel structures enclosing a well-defined cavity that can bind and effect a slow release of volatile pheromones^28^,^46^,^47^,^48^,^49^. There is also a growing body of evidence that MUPs act as signalling molecules in their own right. The pattern of urinary MUPs can act as an individuality signal in competitive interactions^29^,^32^,^36^, in mate choice^30^ and in the maintenance of heterozygosity^33^, while sharing of MUP pattern provides a genetic marker for kin recognition^31^,^37^. Darcin (MUP20), notably a peripheral gene product, is expressed exclusively by males and is inherently attractive to females. Darcin also stimulates associative learning, evoking a persistent remembered attraction to both the airborne volatile odour of the individual male associated with this pheromone and the physical location of the male’s scent marks^34^,^35^,^50^.

Here, we assess the potential that urinary MUPs could be candidates to explain subspecies divergence in mate recognition signals, and preference in the context of reinforcement^27^. As an important first step we provide a phenotypic characterization of this complex molecular signal at the protein level. First, we assessed the extent to which the two subspecies differ (a) in their total urinary protein output, as suggested from a comparison of mice captured from the border of the Bavarian - Czech Republic hybrid zone^39^, and (b) in their qualitative and quantitative expression of specific urinary MUP isoforms, which we further characterise by intact mass analysis and peptide mass fingerprinting to understand the extent of sequence differences between MUPs expressed by the two subspecies. Our study identifies eight MUPs that show qualitative and/or quantitative divergence between the two subspecies. Second, we compared levels of divergence between contact populations (represented by mice sampled in different locations close to the Danish hybrid zone, see Fig. 1 and Smadja *et al*.^23^) and allopatric populations of the two subspecies (represented by mice sampled in different allopatric locations, Fig. 1). Here, we do not aim to test whether reinforcement occurs; other studies involving behavioural and genetic approaches have already demonstrated the plausibility of such mechanisms^11^,^12^,^27^. Instead, we track molecules that show a pattern of divergence consistent with reinforcement: candidate molecules are expected to show higher subspecies divergence among samples from the contact zone than among allopatric samples, as well as character displacement within a given subspecies (i.e. marked differences between contact and allopatric samples of the same subspecies). Our study identifies seven MUPs that correspond to these criteria.

## Results

### Variation in urinary protein output

As expected, strong sexual dimorphism in total urinary protein output was evident in both subspecies (effect of sex on mg protein/mg creatinine to adjust for urine dilution, F_1,135_ = 78.4, p < 0.0001). Male protein output was approximately 3.5 times higher on average than female output, though individual output varied widely between males (Fig. 2a). Overall, *musculus* had slightly higher average protein output than *domesticus* (F_1,135_ = 4.48, p = 0.036), though this subspecies divergence tended to vary according to geographical origin (interaction, F_1,135_ = 3.29, p = 0.07; Fig. 2a). Broken down by origin, subspecies divergence was evident in allopatric samples (F_1,42_ = 8.77, p = 0.005) but not in contact samples (F_1,62_ = 0.06, p = 0.81), rejecting the hypothesis of stronger subspecies divergence in total urinary output in contact than in allopatric mice.

Urine samples from *musculus* were more dilute (had lower creatinine concentration) than *domesticus* samples (F_1,135_ = 24.7, p < 0.0001) regardless of sex or geographical origin (Fig. 2b; all interaction terms p > 0.20). Female samples from both subspecies also had lower creatinine than male samples (F_1,135_ = 9.71, P = 0.002; Fig. 2b). This could be because females had more dilute urine or had lower muscle mass. However, subspecies divergence in urine dilution was not influenced by geographical origin.

By contrast, subspecies divergence in the concentration of urinary protein in a fixed volume of urine (Fig. 2c) was influenced by both sex (subspecies × sex interaction, F_1,135_ = 5.41, P = 0.022) and geographical origin (subspecies × origin interaction, F_1,135_ = 4.01, P = 0.047). This was due to particularly low protein concentration in female *musculus* samples from the contact zone compared to *domesticus* or to allopatric samples of either subspecies (female subspecies × origin interaction, F_1,73_ = 4.08, P = 0.047). For females, this resulted in highly significant subspecies divergence in contact (t_55_ = −4.19, P = 0.0002) but not allopatric samples (t_18_ = −0.14, P = 0.89). No subspecies divergence in protein concentration was evident among male samples (effect of subspecies, F_1,62_ = 1.12, P = 0.29; subspecies × origin, F_1,62_ = 0.71, P = 0.40). Thus, although total protein output did not differ when corrected for the dilution of samples, there was evidence of stronger subspecies divergence in the protein concentration of samples among contact females.

The overall protein composition of urine samples was examined by SDS-PAGE (Fig. 2d). A major band at approximately 21 kDa was predominant in urine of both subspecies and sexes and migrated in the expected position for MUPs^51^. In addition, a higher mobility (lower apparent mass) band was present in 95% of male samples but not in any female samples, migrating at approximately the same position as the atypical MUP darcin^34^,^52^. This marked preponderance of MUPs in urine samples allowed for further molecular characterisation of these proteins. Below, we first establish the difference in MUP masses expressed according to subspecies, sex and origin. We then use peptide mass fingerprinting on electrophoretically resolvable proteins to establish the degree of similarity in MUP sequences between the two subspecies, for as many sequences as feasible. Finally, we examine quantitative differences in expression to look for divergence according to subspecies and origin within each sex.

### Qualitative variation in protein mass profiles

We used electrospray ionization mass spectrometry (ESI-MS) of the desalted urine samples to acquire a profile of protein masses between 18,400 to 19,000 Da. To examine relative intensities of each mass independent of the overall protein concentration of a sample (analysed above), intensity was scaled from 0 to 1 where 1 referred to the most intense peak in that sample. Each group profile presented in Fig. 3 is an averaged spectrum derived from multiple individuals of the same subspecies, sex and geographical origin (contact or allopatric). These profiles were used to identify the commonly occurring masses to direct further characterisation. For *domesticus* from contact populations (Fig. 3a,b), the individual processed mass spectra of all 45 samples could be aligned easily to create an average spectrum for each sex, reflecting consistency in the masses expressed across individuals. Most of the allopatric *domesticus* samples could also be aligned with each other, except for the four samples from Tarbes, which had to be aligned to other allopatric samples based only on the most prevalent peak (18694 Da). The broader, slightly asymmetric peaks evident in the average spectrum for allopatric *domesticus* reflect differences in the masses of other peaks in Tarbes sample profiles (Fig. 3e,f). All *musculus* samples from the three allopatric locations aligned easily (Fig. 3g,h), as did those from the contact locations (Fig. 3c,d).

The ability to discriminate individual MUP masses is limited by the effective resolution of the mass spectrometer, typically ± 2 Da in the processed mass value obtained with this instrument. Notwithstanding this restriction, it is possible to comment on the overall mass-resolved protein profile in each subspecies.

#### M. m. domesticus profiles

Overall, ten distinct mass peaks were evident within *domesticus* averaged profiles. Within contact or allopatric populations, male and female averaged profiles were similar (for contact samples compare Fig. 3a with 3b; for allopatric samples compare Fig. 3e with f). The main sex differences were in the expression of a mass peak at 18646 Da, seen only in males from allopatric populations, and expression of small peaks at 18893 Da or 18897 Da only in males from allopatric or contact populations respectively. The 18646 Da mass corresponds within 1 Da to that of MUP7 in the inbred reference genome (MGI nomenclature is used throughout), where expression is also male specific^42^, while 18893 Da corresponds to the mass of the male-specific MUP20 known as darcin^34^. In addition to these three masses with male-specific expression, four mass peaks correspond within 1 Da to masses encoded by other known *Mups* in the C57BL/6 J reference genome: 18666 (*Mup8* – 18665 Da), 18682 Da (*Mup13* – 18682 Da, *Mup17* – 18683 Da), 18694 Da (*Mups 1, 12*–18693 Da; *Mup2* – 18693 Da; *Mups 9,11,16,18,19*–18694 Da), and 18708 Da (*Mup10*). Two more peaks correspond within 1 Da to masses of known MUPs from *domesticus* derived from Edmonton, Canada^53^: 18651 Da (contact mice only) and 18724 Da. Only the mass peak at 18731 Da (a small peak only observed in allopatric Tarbes males) did not correspond to an established *domesticus* MUP.

#### M. m. musculus profiles

Ten distinct mass peaks were evident within *musculus* averaged profiles. Within contact or allopatric samples, males and females expressed similar peaks but with differences in relative intensities (for contact mice compare Fig. 3c with d; for allopatric mice compare Fig. 3g with h). The main qualitative sex difference was in the expression of a peak at 18893 Da in males only (corresponding to the published mass of darcin). Four peaks had masses that could correspond to those of known central *Mup* genes from C57BL/6J: 18694 Da was consistently expressed in *musculus* samples (a mass encoded within 1 Da by at least eight genes in the C57BL/6J reference genome, see *domesticus* above); 18708 Da was expressed at low level in some *musculus* samples (the mass of *Mup10*); 18713 Da expressed in some allopatric samples (the mass of *Mup14*), while 18711 Da in other samples was within 2 Da of this. Five mass peaks did not correspond to the masses of any currently known *Mup* genes: 18575, 18679, 18731 and 18752 Da were seen in *musculus* samples from contact and allopatric populations, and 18716 Da which was observed only in allopatric *musculus* samples from Poland. We did not observe peaks corresponding to masses 18646 or 18651 Da in *musculus* profiles.

### Protein identification by peptide mass fingerprinting

Protein bands separable by either SDS-PAGE (darcin) or by native gel electrophoresis (central MUPs) were characterised further by peptide mass fingerprinting, analysing peptides that were the result of cleavage after lysine and arginine residues (trypsin) or just lysine residues (endopeptidase LysC). The masses of many of the derived peptides matched cognate peptides from C57BL/6J MUPs, and we used the C57BL/6J protein sequences as a reference for our analysis. The masses of a few tryptic (prefixed T) or endopeptidase LysC (prefixed L) peptides differed from those obtained by digestion of the known C57BL/6J isoforms. We explored whether each peptide mass change could be elicited, most simply, by a single amino acid substitution that might have been observed previously in known MUPs. Secondly, we assessed whether the combination of mass shift in the differing peptide(s) could explain the measured mass of the intact protein; in other words, whether the localized amino acid change(s) could explain in full the mass and thus, by inference, sequence of the new protein. A summary of these findings is presented in Fig. 4 and below, with full details of this analysis in Additional File 1 and Supplementary Figs S1, S2 and S3.

#### Darcin

Almost all male samples from both subspecies (contact and allopatric populations) exhibited a high mobility band on SDS-PAGE that was absent from female samples (Fig. 2d). This band recapitulated the electrophoretic behaviour of the male peripheral MUP pheromone, darcin, identified previously in male laboratory mice^52^ and in wild-derived *domesticus* from the UK^34^,^52^, US^53^,^54^ and Switzerland^55^. While a mass peak at 18893 Da, corresponding to the known mass of darcin, was evident in allopatric male *domesticus* samples (Fig. 3e) and in all male *musculus* samples (Fig. 3c,g), a mass peak that was 4 Da heavier (18897 Da) was evident across all male *domesticus* contact samples (Fig. 3a). Nonetheless, in-gel digestion of the high mobility SDS-PAGE band in all males confirmed that all the peptides matched the peripheral MUP darcin (Supplementary Fig. S1). The 4 Da shift in mass in male *domesticus* in contact populations suggests minor amino acid change(s) but this could not be detected by peptide mass fingerprinting, suggesting that the change(s) occurred in peptides that were too small to detect.

#### M. m. domesticus central MUPs

Three native gel bands were resolved in both male and female contact *domesticus* (Fig. 4a, labelled A–C). The peptides generated by digestion of each band (details in Supplementary Fig. S2) showed a very high degree of matching to those from known MUPs encoded by central *Mup* genes, which differ from each other in only a small number of amino acids. A survey of known central MUP sequences^42^,^53^ reveals a restricted set of known amino acid substitutions at only twelve locations in the sequence of 162 residues. Analysis of tryptic peptides from *domesticus* gel bands highlighted the occurrence of many of these positional variants.

*M. m. domesticus* band A (both sexes) contained peptides that would yield MUP sequences of mass 18693 Da (a sequence corresponding to MUP1 and MUP12) and 18682 Da (a new combination of previously observed substitutions). Both of these predicted MUP masses were observed in the corresponding intact mass profiles (Fig. 4a). The combined peptide mass fingerprints of *domesticus* band B (both sexes) aligned strongly with MUP10 (18708 Da) and no mass shifted peptides were evident (Fig. 4a). From this, it is very likely that band B contained MUP10.

The combination of peptides in band C (both sexes) gave a predicted protein mass of 18683 Da, corresponding closely to the observed mass at 18682 Da (Fig. 4a). This predicted sequence corresponds to a known MUP sequence recently reported in wild *domesticus* from Canada^53^. However, an additional peptide observed in band C also indicated the presence of another MUP that had a different dipeptide sequence (D_34_N_35_) from the 18682/3 Da MUP (peptide L4 in band C, Supplementary Fig. S2). Notably, the major peak at 18651 Da observed in intact mass analysis of *domesticus* urine from contact populations had not been explained by this analysis. This mass does not correspond to any *Mup* genes known from laboratory mice, but Sheehan *et al*.^53^ recently reported a new sequence from *domesticus* trapped in Canada with a predicted and observed intact mass of 18651 Da. This new sequence is consistent with the peptides observed in male band C, including the D_34_N_35_ dipeptide sequence that had not been accounted for, except that we did not observe a peptide corresponding to R_161_L. As this change occurs in the penultimate residue of the MUP sequence, it could be difficult to detect by peptide mass fingerprinting. Purification of this protein and detailed molecular analysis would be required to confirm whether the 18651 Da mass in contact *domesticus* corresponds to the new sequence reported by Sheehan *et al*.^53^, but this could not be achieved with the samples obtained for this study.

#### M. m. musculus central MUPs

Two major bands were resolved by native gel electrophoresis in both male and female *musculus* (Fig. 4b, bands D & E). Peptides observed from male band D (intensely stained) were highly consistent with those commonly observed in *domesticus* MUPs, with one exception (Supplementary Fig. S3). One trypic peptide (T9) involved a 22 Da mass shift that would be consistent with an Asp to His amino acid change. This substitution has not previously been reported in any *domesticus* MUP sequence (nor any other variation in the T9 peptide). The combination of observed peptides would yield a protein of predicted mass 18752 Da, exactly consonant with the intact mass peak of 18752 Da observed in male *musculus* (Fig. 4b). A band of the same mobility in female *musculus* was too weak for peptide mass fingerprinting, consistent with much lower expression of 18752 Da among females.

Peptides from *musculus* band E in both sexes mapped well to the 18694 Da MUP that is encoded by five genes in C57BL/6 (*Mup9, Mup11, Mup16, Mup18, Mup19*), and there was a corresponding peak at 18694 Da in intact mass profiles (Fig. 4b). In addition, a single mass shifted peptide (T6, Supplementary Fig. S3) was exactly consonant with the main protein peak of 18731 Da evident in intact mass profiles of male and female *musculus* (Fig. 4b). This new sequence could be explained as a new combination of substitutions previously observed in *domesticus* MUPs.

The substantial similarity in peptides between individual isoforms means that not all sequences that represent different combinations of the same set of peptides can be confidently identified and quantified using this approach, and not all of the MUP masses observed as low intensity peaks in intact mass profiles could be confirmed by peptide mass fingerprinting. However, it was clear that novel proteins, in either *musculus* or *domesticus*, could be generated by new combinations of previously observed amino acid changes^42^,^53^. Nonetheless, the 18752 Da mass in *musculus* must be a novel protein sequence that is not explained by combinations of known amino acid substitutions in *domesticus*.

### Quantitative variation in protein mass profiles

While we were able to identify some new central MUP sequences using peptide mass fingerprinting, the substantial similarity between central MUP isoforms means that it was not possible to define the full set of MUP isoforms expressed by each individual and quantify expression at this level. Instead, to examine quantitative variation in expression according to subspecies, sex and geographical origin, we measured for each individual sample (n = 137) the relative intensity of each of the main mass peaks (above), expressed as a proportion of the summed intensity of all central MUP peaks in that sample. Note that peaks that differ by at least 2 Da clearly represent different MUPs, but we cannot know whether the same mass peak consists of the same isoforms across samples. Nonetheless, a quantitative difference in intensity of the same mass peak between groups indicates a difference in expression, whether or not the isoforms are the same. As this approach would tend to underestimate differences if isoforms of similar mass were not the same between subspecies or origin, our analysis provides a conservative measure of phenotypic variation. This analysis was restricted to central MUP isoforms, as their relative expression can be measured reliably from intact mass profiles^53^. Although the peripheral MUP darcin was detected in male samples by intact mass analysis, its unique structure meant that it yielded weaker signals on the instrument used in this study than expected from the amount evident on SDS-PAGE gels, and we could not reliably quantify its expression levels using this approach.

When the entire set of individual expression intensities for central MUP masses was subjected to hierarchical clustering (Fig. 5), there was clear separation between *musculus* and *domesticus* profiles at the highest level. Beneath that, although less marked, there was a segregation of contact and allopatric profiles. The separation between male and female mice within subspecies and origin was much less clear based on these central MUP masses. There was also no segregation of samples from the same trapping location.

To identify candidates for assortative mating, we looked for mass peaks that differed in expression between the two subspecies, so might provide a basis for subspecies signals and preference divergence (Fig. 6, Table 1). To identify candidates for reinforcement, we looked for a difference in expression between the two subspecies that was greater in contact than in allopatric samples (Table 2). Proteins that were not expressed by either of the two subspecies in contact samples are not considered further in this analysis (18646 Da, 18716 Da and 18713 Da mass peaks). The frequency of occurrence of each mass by subspecies, sex and origin is given in Supplementary Table S1.

Three masses were evident in both contact and allopatric *musculus* samples but were not detected in any *domesticus* samples (18575, 18679, 18752 Da). Contingency chi-squared tests confirmed that the frequency of detection of a clear peak in *musculus* differed significantly from the absence of detection in *domesticus* samples for each of these masses (Table 1). Potentially, expression only by *musculus* could be used to discriminate *musculus* from *domesticus*. The mass 18679 Da showed significantly stronger expression in allopatric than in contact samples, while 18752 Da levels were very similar between contact and allopatric samples in both sexes (Table 2). Only 18575 Da was present at higher intensity in contact compared to allopatry, although the relative amount of this newly observed mass was very low in all samples (Fig. 7).

Two other masses (18711 and 18731 Da) were present in *musculus* but not *domesticus* in contact samples. The first, a minor peak at 18711 Da, was uncommonly present in allopatric samples of both subspecies (15% *musculus*, 24% *domesticus*), with no subspecies difference in expression level (Table 1). However, 18711 Da was exclusive to *musculus* in contact samples (in 85% male and 42% female samples, Table 1), with a bias in level between subspecies that was significantly stronger than in allopatric samples (Table 2; Fig. 7). The second peak at 18731 Da was also exclusive to *musculus* in contact samples (in this case a major peak that was expressed in 100% of all *musculus* samples), and was only rarely expressed among allopatric *domesticus* (from a single locality, Tarbes, Fig. 5 and Supplementary Table S1 which shows frequencies of expression of each mass peak). The bias in expression by *musculus* was much stronger in contact compared to allopatric samples (Table 2, Fig. 7).

Three masses in contact samples were evident only in *domesticus* (18651, 18682, 18724 Da); another was observed only in *domesticus* among contact samples but in both subspecies among allopatric samples (18666 Da). The 18651 Da mass was consistent with a potential reinforcement candidate (Table 2, Fig. 7): it was exclusive to *domesticus* contact samples, where it occurred at high frequency in both females (97%) and males (87%), contrasting strongly with the lack of expression in either *musculus* contact (Table 1) or *domesticus* allopatric samples (Table 2). The 18666 Da mass was present at much lower level than the 18651 Da peak, but still with a stronger bias between subspecies in contact samples, while this was not the case for 18682 or 18724 Da (Table 2).

Two masses that were very common in both *musculus* and *domesticus* samples from allopatric and contact populations, were present at different levels according to subspecies and origin. A mass peak at 18694 Da was evident in almost all samples. Its proportion in each profile was substantially higher among *domesticus* compared to *musculus* in allopatric samples, a subspecies difference that was considerably reduced in contact samples and possibly even reversed slightly among females (Table 2, Fig. 7). By contrast, the relative amount of the commonly observed mass at 18708 Da in *domesticus* profiles was substantially higher in contact than in allopatric samples but was rare and only a small peak in *musculus* (Figs 6 and 7; Table 2).

## Discussion

Major urinary proteins are involved in social and sexual communication in the house mouse^56^, and we hypothesised that they could participate in signal divergence underlying assortative mate preference and possibly speciation by reinforcement between its two European subspecies^11^,^27^,^57^. Earlier behavioural investigations have shown that contact populations (particularly *musculus*) discriminate between urine of the two subspecies, whether urine is of contact or allopatric origin^11^,^12^. This indicates that signal divergence between the two subspecies took place in allopatry and that contact mice have the ability to discriminate between these subspecific signals. Additional studies comparing the extent of urine scent divergence between the two subspecies reveal that discrimination is more marked when urine is from contact mice, suggesting that signal divergence between the two subspecies may also be more marked in contact populations^57^. These findings motivated us to look for two types of candidates: those showing divergence both in allopatry and contact, and those showing a pattern of reinforcement (i.e. character displacement between contact and allopatric urine of the same subspecies). The present study reveals distinct patterns of MUP expression in *domesticus* and *musculus*, and identifies potential candidate MUPs that could have been recruited for assortative mating, either during divergence in allopatry or during a process of reinforcement in the hybrid zone. Our results underline the diversity of MUPs expressed in wild populations of the house mouse, and allow us to describe the MUP profiles of *M. m. musculus* for the first time.

The number of protein masses that we identify as candidates that could contribute to assortative mating is relatively large (around eleven different mass peaks that could represent eleven or more different MUP isoforms). If most or all were confirmed to be involved, this complexity would contrast with the relative simplicity of signals allowing assortment that have been identified in a limited number of species, e.g. moths of the genus *Ostrinia* in which different combinations of E and Z isomers of two molecules allow sex discrimination between eight distinct species e.g. ref. 58. Notably, though, this complex of major urinary proteins in the house mouse is encoded by a single cluster of genes, inherited together as a haplotype, that provides sufficient polymorphism at the individual level to be used as a signal for individual and kin recognition^30^,^37^,^53^. These roles in individual and kin recognition imply relatively high between-individual and population variation, which arises through a combination of variation at amino acid coding sites and differential transcription of central *Mup* genes across individuals^42^,^53^. To function simultaneously as a signal for subspecies recognition, dual selection on the MUP profile may favour both individual distinctiveness (polymorphism) and similarity (salient traits shared by individuals of a given group, here subspecies). Our study shows that despite variation within each subspecies, individuals of the same subspecies share MUP profiles that have marked similarities (i.e. expression of sets of the same MUP isoforms and/or similar levels) and might have the potential to convey subspecific identity (Fig. 5). Most mass peaks detected in our samples were expressed by only one of the two subspecies. However, we cannot tell from this relatively limited sample whether these represent MUPs that are exclusive to each subspecies or are expressed at much greater frequency and thus much more likely to be sampled in one subspecies (see also ‘Molecular characteristics of candidates’ below). While we also cannot be sure whether mass peaks that were shared represent identical MUP isoforms in the two subspecies because of the technical difficulty of resolving such similar isoforms, evidence from peptide mass fingerprinting suggests that at least two MUPs are shared: *musculus* expressed darcin (encoded by *domesticus Mup20*) in addition to the commonly observed 18694 Da MUP encoded by multiple genes in the *domesticus* reference genome (identical mature primary sequences encoded by *Mup9, 11, 16, 18, 19*).

### Subspecies divergence

Eight MUP mass peaks showed differences between the subspecies both in allopatric and contact urine samples, suggesting that some divergence took place in allopatry. Four of these masses were more strongly or exclusively expressed in *musculus* (18575, 18679, 18731, 18752 Da) while four were more strongly or exclusively expressed in *domesticus* (18666, 18682, 18708, 18724 Da). MUP isoforms within these mass peaks (each peak containing at least one isoform) are potential candidate molecules that could underlie the assortative preference shown by Danish contact mice towards allopatric urine from the two subspecies^12^. If all or some of these MUP candidates are involved in mate signalling, selection in the contact zone could have favoured the evolution of assortative discrimination and preference (shown by contact but not allopatric mice of the two subspecies^11^). Contact mice of each of the two subspecies could respond exclusively to MUPs of their own subspecies, or to both types of MUPs, accepting the former and rejecting the latter. It may be possible to test this in a follow-up study by manipulating mouse urine with biosynthesized MUPs once the sequences of these candidate MUPs are fully resolved. Such an approach has been applied to phytophagus insects showing that, for example, preference of the European corn borer for maize may actually hide strong avoidance of mugwort^59^, while in *Rhagoletis pomonella* flies, both attraction to the preferred host and avoidance of other hosts are involved in race isolation^60^.

Although MUPs have diverged between the subspecies, it is still necessary to show that mice discriminate such differences. It is known that mice can distinguish similarity or difference in MUP profiles for individual^29^,^30^,^36^ or kin recognition^37^, though so far the ability to discriminate has been examined only in the context of ‘same’ versus ‘different’ MUP profiles within a subspecies. An ability to discriminate between ‘different’ MUP profiles according to their degree of difference to own or locally familiar profiles has yet to be tested.

### Some MUPs are potential candidates for reinforcement

Our study demonstrates stronger subspecies differences in expression of some of the MUPs in our contact compared to allopatric samples, which are consistent with findings from behavioural bioassays^57^, and predictions on reinforcement. These candidates are mass peaks that differed between subspecies in both allopatric and contact samples but showed stronger differences in contact samples than in allopatry (18575, 18666, 18708, 18731 Da), or differed between the two subspecies only in contact samples (18711 Da expressed only by *musculus* in contact and by both in allopatry; 18651 Da seen only in *domesticus* contact samples and not in *musculus*). Among these peaks, the newly identified 18731 Da MUP was the only one detected in every *musculus* sample. Further, this mass clearly dominated the profiles of *musculus* from the contact zone (i.e. had the highest relative intensity, especially among males), while it was also an important peak but not always the peak of highest relative intensity in allopatric *musculus* MUP profiles. By contrast, this mass peak was absent from all contact *domesticus* samples and was rarely observed among the allopatric *domesticus* samples. Although the mass peak at 18651 Da was exclusive to *domesticus* contact samples in this study, a MUP of this mass has been reported in allopatric *domesticus* from Canada^53^ and we quite frequently observe a similar mass peak in allopatric *domesticus* from the UK (JLH, RJB, AJD, GGB unpublished data).

Although we could not quantify darcin expression in this study, the relative intensity of the fast-running band on SDS-PAGE suggested that levels of expression were of similar magnitude among males in the two subspecies (see Fig. 2d). Evidence from intact mass and peptide mass fingerprinting indicate that the darcin sequence expressed by *musculus* is the same as that expressed by allopatric *domesticus*, consistent with strong conservation of a male sex pheromone that has important roles in stimulating attraction to males scents, associative learning and neurogenesis^34^,^35^,^50^,^61^, as well as triggering competitive and defensive aggression against intruder males^36^,^38^. Nonetheless, we observed a novel difference in the mass of darcin in contact *domesticus* samples, although further work is needed to identify the specific changes to the sequence. As this difference was observed only in contact samples, this provides another candidate for reinforcement. Darcin could undoubtedly be critical for induced learning of spatial cues and individual identity that would help maintain the subspecies boundaries. However, as darcin is expressed at functionally relevant levels only in the urine of males, such divergence would only influence female sexual preference.

For central MUPs, we found consistent differences in MUP profiles according to subspecies and origin among males and females (see Table 2). Thus, if central MUPs are involved in subspecies signalling, both males and females of contact populations could base assortative preferences on a similar combination of MUPs, albeit with some individual and sex-specific variation in the relative concentrations of the different masses. Notably, though, female but not male urine showed a highly significant subspecies divergence in total urinary protein concentration in contact samples, due to the particularly low concentration of protein in *musculus* females. As urinary protein concentration is sexually dimorphic, it is possible that it could contribute to mate discrimination between the subspecies^39^.

### Molecular characteristics of candidates

While detailed analysis of the different MUP isoforms has previously focused on those expressed by *domesticus*, our intact protein analysis has revealed new masses in *musculus* samples that do not correspond to known isoforms from *domesticus*. Further, peptide mass fingerprinting revealed three new central MUP sequences not previously reported, including two that match predominant mass peaks in the urinary MUP profiles of *musculus*. Importantly though, all but one of the central MUP sequences reported here can be explained by mass shifted peptides that also occur commonly in other MUPs known from *domesticus* laboratory or wild-derived mice. Only the newly identified 18752 Da MUP, very commonly expressed in *musculus* but with highest levels among males, required a novel substitution. However, even this mass would be most readily explained by a single novel amino acid change within a sequence of commonly observed peptides, typical of the very high similarity found among all central MUP sequences.

Two recent studies^62^,^63^ attempted to quantify expression of different MUP isoforms among wild male *musculus* derived from Austria, but failed to find any of the differences between *musculus* and *domesticus* reported here, including the new sequences that dominated *musculus* profiles in both contact and allopatric samples in our study. However, the approach used to identify unknown MUPs expressed by *musculus* in these studies involved digesting all proteins in a urine sample and required matching the resulting peptides to MUP sequences predicted by *Mup* genes from the laboratory mouse reference genome (derived from *domesticus*). The assumption was made that matching peptides belonged to these laboratory mouse genes. Such an approach would fail to identify any ‘new’ sequences that were not present in the reference genome. Further, as central MUPs consist of different combinations of a limited set of amino acid changes (resulting in the same sets of peptides)^42^,^43^,^53^, such an approach cannot be used to identify or quantify central MUP expression in wild mice where the combinations (i.e. *Mup* gene sequences) are unknown. Our intact mass analysis, which measures a property of the intact MUP rather than fragments, combined with identification of new sequences through peptide mass fingerprinting, overcame these difficulties.

Changes in central MUP isoforms may be detected as differences in the proteins themselves detected through vomeronasal receptors (e.g. ref. 36) or as differences in the profile of volatiles bound and released from the central cavities of MUPs^52^,^64^,^65^,^66^. The cavity residues of central MUPs are highly conserved, with only one out of 20 residues showing variation. This involves either a valine or phenylalanine variation at residue number 56, located at the base of the central cavity^49^. The dominant isoforms that differed between *musculus* and *domesticus* MUP profiles in the contact zone appear largely to concern MUP surface rather than cavity residues, with the dominant MUPs in both subspecies sharing a valine residue at cavity position 56. Further analysis of more minor masses will be needed to confirm whether there are any differences in the ratio of valine to phenylalanine residues in the cavity that might influence volatile ligand binding differently in the two subspecies. Importantly though, isoform-specific differences in MUP surface residues should be detectable through vomeronasal V2R receptors when animals make direct nasal contact with urine^36^. The restricted number of sites that show variation, further confirmed in this study across both subspecies, suggests that MUP variation may be constrained by interactions with these vomeronasal receptors^53^.

## Conclusions

By pinpointing candidates that could be biosynthesized for use in bioassays of mate preference, our study creates new opportunities to understand the evolution of mate recognition at a molecular level. In particular, we will be able to test whether these molecules are involved in reproductive isolation, the relative importance of signal divergence in allopatry versus in contact for assortative preference, and whether there is sexual dimorphism in these signals and response. Further, characterising the molecular phenotype of assortative mating will help to identify its genetic basis and, as a consequence, address the evolutionary mechanisms involved. The established functions of MUPs in *domesticus* as identity signals and as pheromones used in competitive, cooperative and sexual communication suggest that at least some of the subspecies differences revealed are likely to be shaped by natural and/or sexual selection, whether directly or indirectly.

Moreover, in the framework of discussions on the concept of “recognition at group boundaries”^67^,^68^,^69^,^70^, our study opens opportunities to characterize the nature of a putative species recognition signal. Identifying and validating the molecules involved in assortative preference may help to better define the concept of species recognition by addressing difficult questions such as: are signals involved in assortative mating between incipient species exclusively used in species recognition contexts, or do they also participate in sexual and/or social communication within species? Does species discrimination during reproductive interference and competitive interference rely on the same signals^71^,^72^?

## Methods

The protocols used to gain the urine samples used in this study were approved by the Direction départementale de la protection des populations, France, to GG (authorization no. C34-265). Animal use was in accordance with EU directive 2010/63/EU.

### Urine donors

**Figure 1:**
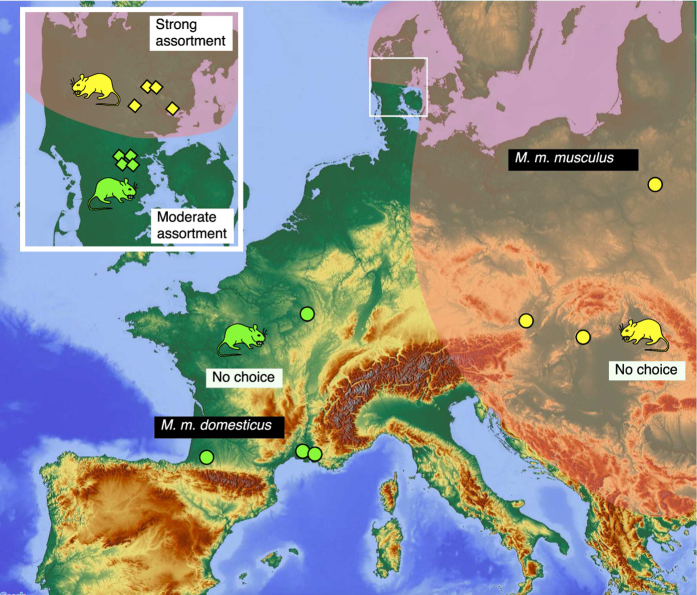
Geographical distribution of the two subspecies of house mice in Europe. The sampling locations of allopatric mice (filled circles) or contact mice (diamonds, shown in boxed area in Jutland) are shown for each subspecies (green: *domesticus;* yellow: *musculus –* the same colour scheme is used for all figures). The map was downloaded from http://maps-for-free.com/ (© OpenStreetMap contributors). The cartography in the OpenStreetMap map tiles is licensed under CC BY-SA (www.openstreetmap.org/copyright). The licence terms can be found on the following link: http://creativecommons.org/licenses/by-sa/2.0/.

**Figure 2:**
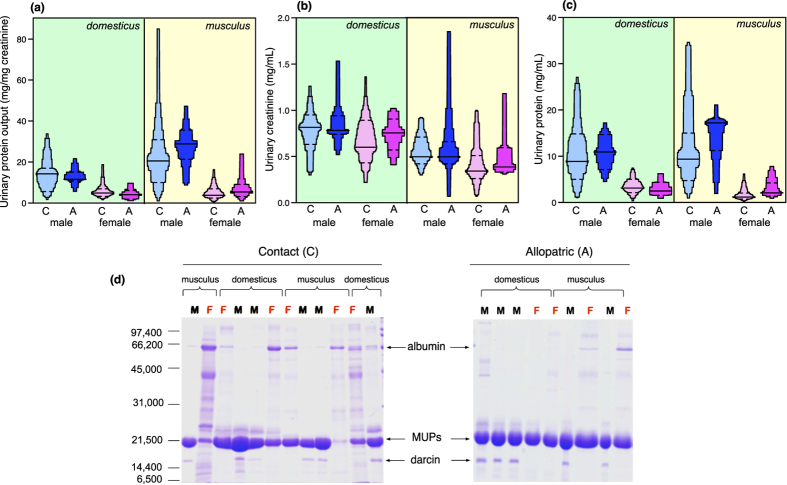
MUP and total urinary protein output. (**a**) Total urinary protein output corrected for urine dilution, (**b**) creatinine as a measure of urine dilution, and (**c**) uncorrected urinary protein concentration for male and female adults of *musculus* and *domesticus* from contact (C) or allopatric (A) origin. (**d**) A representative set of samples resolved by reducing SDS-PAGE, with standard molecular weight markers indicated on the left of the gel. Sample sizes: *domesticus* male contact (n = 22), male allopatric (n = 13), female contact (n = 26), female allopatric (n = 8); *musculus* male contact (n = 18), male allopatric (n = 13), female contact (n = 31), female allopatric (n = 12).

**Figure 3:**
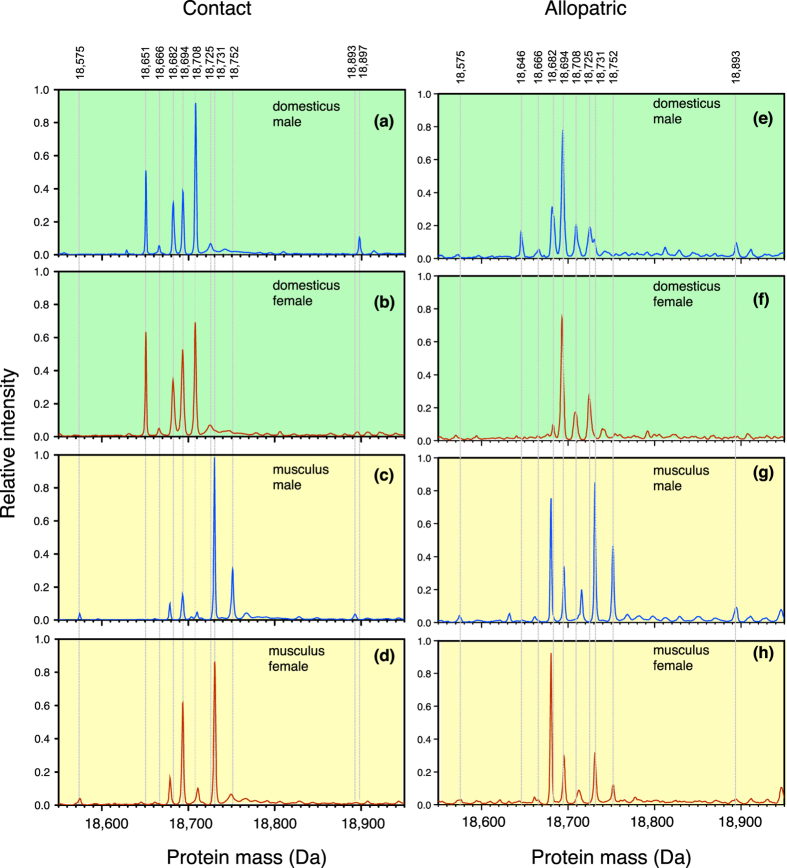
Intact mass profiling of central MUPs according to subspecies, sex and origin. Overall profiles of central MUPs (analysed by electrospray ionisation mass spectrometry, ESI-MS), normalised to scale 0 to 1 and averaged across individuals of the same subspecies, sex and origin (contact or allopatric). Mass peaks shared by multiple individuals are indicated by grey dashed lines, with the mass labelled at the top of the figure. The peak height of darcin (18893 and 18897 Da) is reduced because it does not produce a strong signal on the instrument used for this analysis (see text).

**Figure 4:**
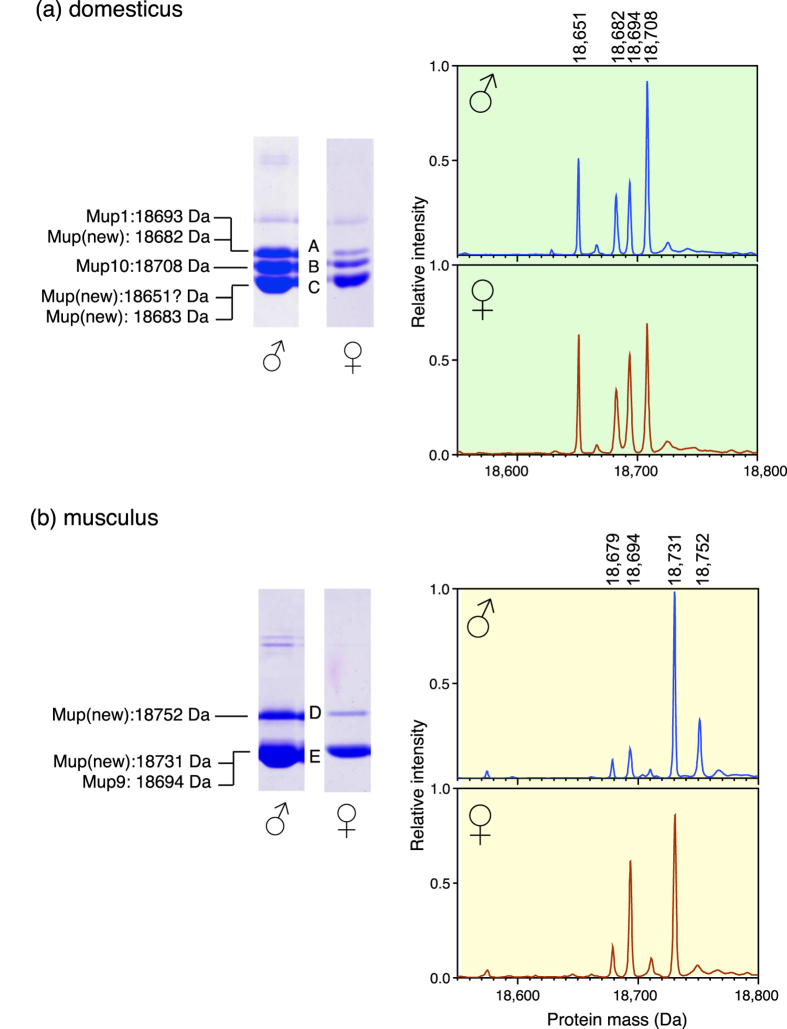
Molecular analysis of MUPs from *Mus musculus domesticus* and *Mus musculus musculus*. Example urinary MUPs from both sexes of (**a**) *domesticus* and (**b**) *musculus* resolved by native gel electrophoresis, with average intact mass profiles (derived by ESI-MS) presented to the right of gel images. Each electrophoretically resolvable band (labelled A through E) was assigned to one or more MUPs by in-gel digestion, followed by peptide mass fingerprinting by MALDI-TOF mass spectrometry (see Supplementary Figs S2 and S3). The intact mass of each of these MUPs was also confirmed as present in the intact mass profiles.

Urine was obtained from adult mice of contact and allopatric origin of the two subspecies (Fig. 1). Mice from contact origin (*domesticus*, 15 males, 30 females; *musculus*, 19 males, 33 females) were captured in June 2000 in eight different locations at the northern (*musculus*) and southern (*domesticus*) edges of the hybrid zone in Denmark (Jutland) and subsequently held under standard conditions in captivity at the facilities of University of Montpellier (location, genotyping and housing details provided in Smadja *et al*.^23^). Allopatric *domesticus* were caught in different locations in France in 2007: Arles, (43°30′0″N, 4°40′0″E; two females, four males), Montpellier, (43°36′0″N, 3°52′60″E; three females, three males), Wassy in the Haute Marne, (48°30′0″ N, 4°57′0″ E; two females, two males) and Tarbes, (43°13′60″N 0°4′60″E; one female, three males). Wild caught (in 2007), or wild-derived allopatric *musculus* were obtained from three locations: Austria (‘Vienna’, 6 females, 5 males, wild and wild derived 2nd generation), as well as Hungary (‘MH’, two females, four males) and Poland (‘MPB’, four females, four males) from two outbred wild derived strains obtained from the wild mice genetic repository (Institute of Evolutionary Sciences UMR 5554, Montpellier, France).

### Urine collection

Urine was collected following either spontaneous urination when handled, or provoked after exerting a gentle pressure on the mouse bladder. The urine was collected in Eppendorf tubes kept on ice during the collection procedure. Urine was sampled repeatedly at different times of the day and different days from a given donor and pooled to produce a single urine sample. All samples were frozen at −20 °C then kept at −80 °C until further biochemical analysis.

### Urine analysis

Urinary protein concentration was assayed using the Coomassie Plus^®^ protein assay (Pierce, Rockford, USA). As urine concentration varies according to the hydration status of the donor at the time of sampling, we introduced the value of using urinary creatinine to standardise urinary protein concentration^51^,^56^,^73^. Urinary creatinine concentration was measured by Diagnostic Creatinine Assay (Sigma, Poole, UK) (see ref. 51 for details). This allowed us to assess urinary protein output as mg/mg creatinine, and examine whether any differences in urinary protein concentration were due to differences in urine dilution or to a difference in protein output *per se*. Protein and creatinine data are provided in Supplementary Table S2. SDS-PAGE analysis of urine was according to ref. 74, using a tris-chloride/tris glycine discontinuous buffer system under reducing conditions. Native gel electrophoresis^42^ was used to resolve charge variants of MUPs.

### Electrospray ionization mass spectrometry

All ESI-MS^75^ was undertaken on a Q-ToF Micro mass spectrometer (Waters, Manchester, U.K.) in positive ion mode. All data sets were processed at 0.25 Da/channel over a mass range of 18,400–19,000 Da. Peak intensities in processed mass spectra were scaled (0–1) to the most intense peak in each sample profile, and profiles of individual samples aligned using SpecAlign^76^ prior to comparison and analysis – this step overcomes minor variation in mass calibration and drift in the mass spectrometer. Once the mass peaks that were consistently expressed across several samples were identified (eliminating any noise in individual spectra), we calculated the relative intensity of each of these peaks as a proportion of the summed intensity of all central MUP peaks in that sample. These data are provided in Supplementary Table S3.

### Peptide mass fingerprinting

In brief, MUPs, resolved by SDS-PAGE or by native gel electrophoresis were subjected to in-gel digestion and the masses of the result peptides generated by either trypsin or endopeptidase Lys-C (‘LysC’) digestion were obtained by high resolution MALDI-ToF mass spectrometry^77^. MGI nomenclature for individual MUPs is used throughout (http://www.informatics.jax.org/searchtool/Search.do?query=mup*).

### MALDI-ToF mass spectrometry

Analysis of peptides from in-gel digests was undertaken using a MALDI-TOF reflectron mass spectrometer (Waters, Manchester, UK) in positive ion mode.

### Data analysis

General linear models examined the fixed effects of subspecies, sex and geographical origin (contact/allopatric) on total urinary protein output (mg/mg creatinine), urinary protein concentration and urinary creatinine concentration (data for n = 143 samples). These response variables were first log transformed to meet the assumptions of parametric analysis (normality of residuals confirmed by Shapiro-Wilks, p > 0.05 after sequential Bonferonni). Intact mass profiles (n = 137 samples) were aligned for each subspecies, sex and origin (contact/allopatric) to identify the mass peaks consistently expressed across multiple samples before calculating an average profile for each group in SpecAlign. For central MUPs, the ESI/MS peak heights of each of the masses were then summed to calculate the expression of each peak in a sample profile as a proportion of total central MUP output. We used contingency chi-squared exact tests to assess differences in the frequency of expression of a particular mass between groups when the mass was absent in one of them (presence was recorded for peaks that were at least 5% of highest peak in that profile to avoid any baseline noise). Where two groups shared the same mass peak, we compared the strength of expression within the profile (expressed as a proportion of total central MUP output) either between two groups using Mann-Whitney U tests, or used a specific contrast for the interaction between subspecies and sample origin in a nonparametric 2 × 2 factorial analysis to assess the hypothesis of a greater subspecies difference in contact versus allopatric samples^78^,^79^. All tests were two-tailed, except where indicated to test the directional hypothesis of greater subspecies difference in contact versus allopatric samples. The alpha thresholds values were adjusted when necessary to control for multiple testing following the Bonferonni sequential method^80^.

**Figure 5:**
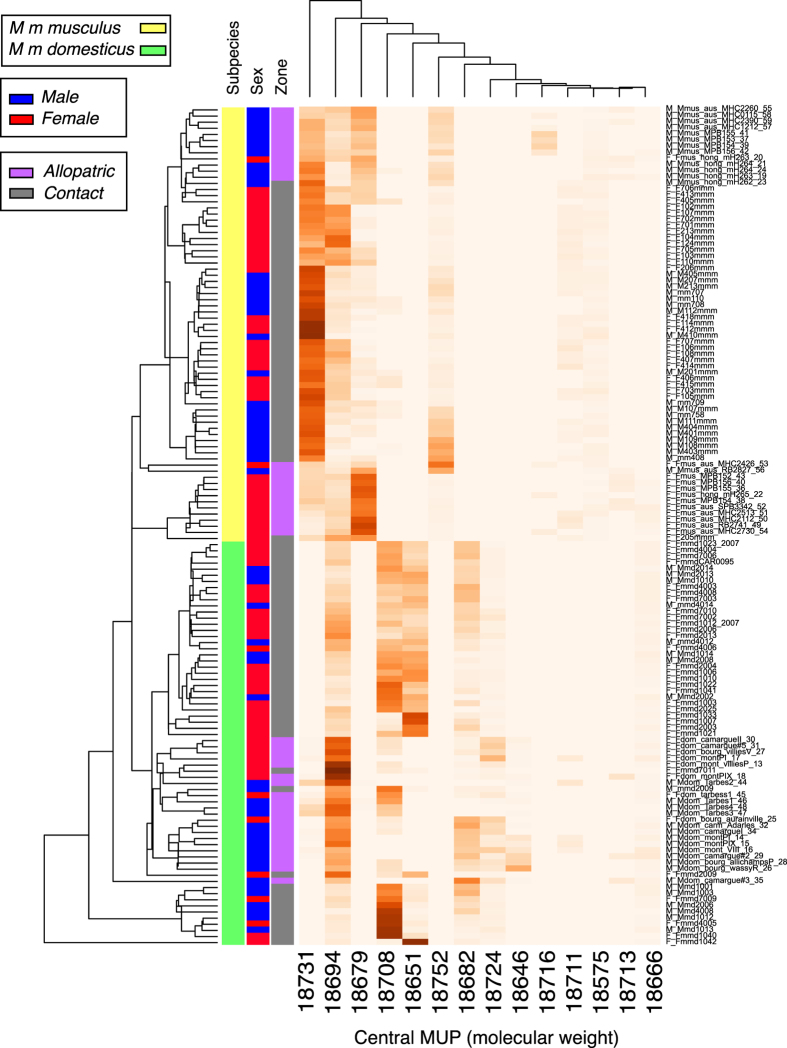
Hierarchical clustering analysis of individual intact mass profiles. Heatmap based on abundances of each central MUP mass, calculated as a proportion of the total intact mass urinary MUP profile for each individual urine sample (n = 137). The data set (protein: rows, individual animals: columns) was subjected to hierarchical clustering using the GMD package (ver 0.3.3, function heatmap 3) in R. Euclidean distances were calculated and median method applied for clustering and generating trees for both proteins and samples.

**Figure 6:**
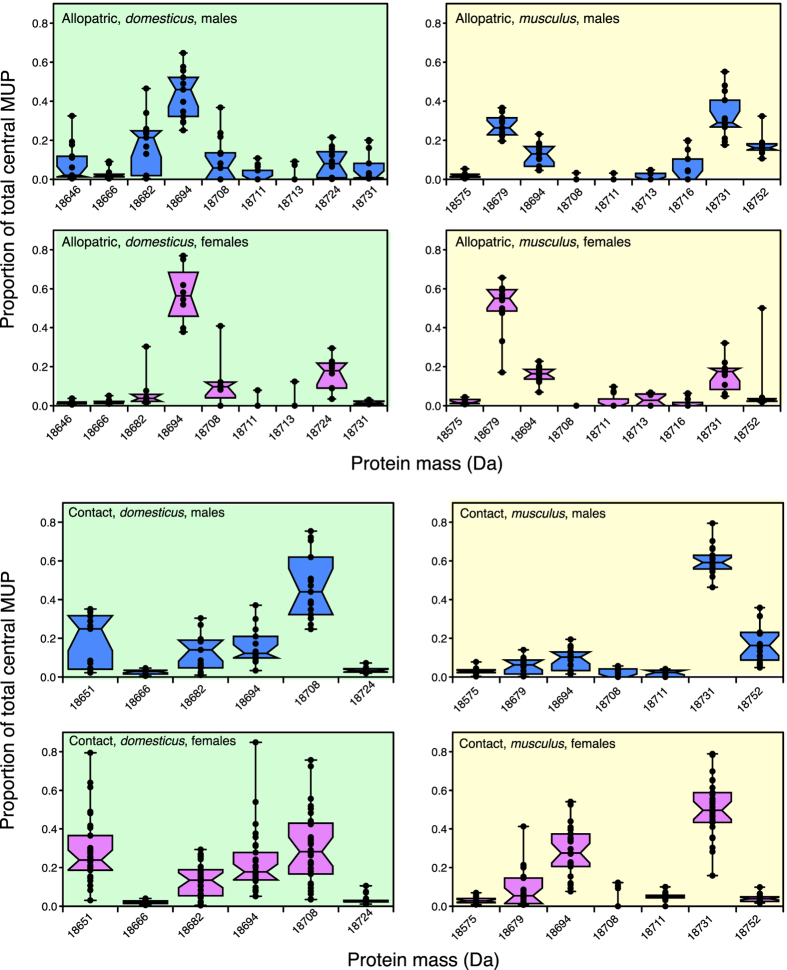
Quantitative profiling of central MUPs. For each individual sample within each group (contact/allopatric, *domesticus/musculus*, male/female), each peak within the intact mass profile is expressed as a proportion of the total of all central MUP peaks in the profile. Individual values are present as black circles, superimposed on a box and whiskers plot (showing medians, 25–75% and furthest ranges). Statistical tests of differences in expression for each mass are shown in Tables 1 and 2. Sample sizes: *domesticus* male contact (n = 15), female contact (n = 30), male allopatric (n = 13), female allopatric (n = 8); *musculus* male contact (n = 19), female contact (n = 27), male allopatric (n = 13), female allopatric (n = 12).

**Figure 7:**
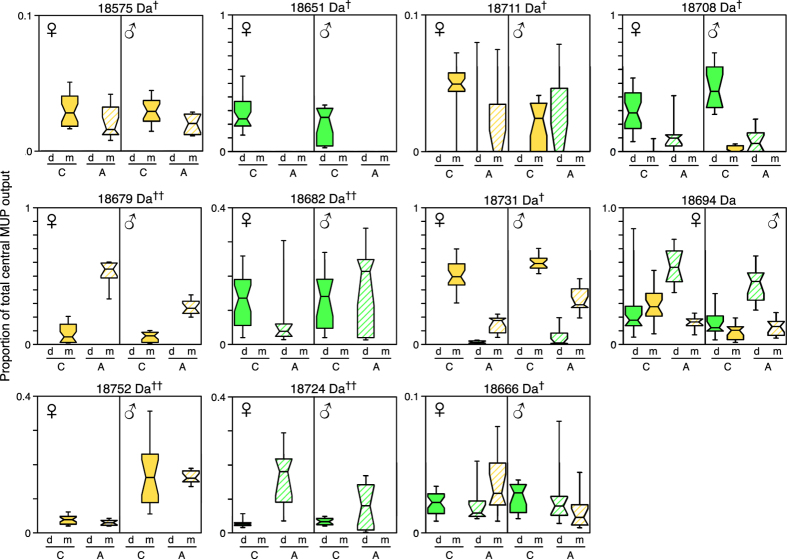
Subspecies differences in contact versus allopatric samples. Box and whisker plots (showing medians, 25–75% and 10–90% ranges) compare expression levels of each mass peak, as a proportion of the total of all central MUP peaks in that sample, for each mass that showed a significant difference in expression between subspecies (statistical tests shown in Table 1). C: contact samples (solid fill), A: allopatric samples (hatched fill); d: *domesticus* (green), m: *musculus* (yellow). ^†^Candidates for reinforcement; ^††^candidates for assortative mating (statistical tests shown in Table 2).

Each subspecies was represented by four contact populations (population = mice trapped in several nearby farms) and 3 distinct and distant allopatric populations (some of which originated from laboratory colonies). Sample size was mostly unbalanced and sometimes too small to allow population to be included as a factor in the analyses described above. Nevertheless, the cluster analysis (Fig. 5) indicates that variation between populations within each category (allopatric or contact) was smaller than differences between categories or subspecies.

R statistical software (v.3.1.0) (R Core Team 2014) was used for the cluster analysis and construction of a heatmap (the R script is provided in Supplementary Information), using the heatmap.3 function of the GMD (0.3.3) package. The software in Barnard *et al*.^79^ was used for nonparametric factorial ANOVAs, and SPSS version 22 (IBM software) was used for all other analyses.

## Additional Information

**How to cite this article:** Hurst, J. L. *et al*. Molecular heterogeneity in major urinary proteins of *Mus musculus* subspecies: potential candidates involved in speciation. *Sci. Rep.* **7**, 44992; doi: 10.1038/srep44992 (2017).

**Publisher's note:** Springer Nature remains neutral with regard to jurisdictional claims in published maps and institutional affiliations.

## Supplementary Material

Supplementary Information

Supplementary Dataset 1

Supplementary Dataset 2

Supplementary Dataset 3

## Figures and Tables

**Table 1:** Subspecies differences in expression^†^ of each mass peak by sex and origin (contact/allopatric).

Mass peak	Female contact	Female allopatric	Male contact	Male allopatric
18575 Da	X^2^ = 20.62, p < 0.0001*musculus only(52%)	X^2^ = 3.33, p = 0.12*musculus* only(33%)	X^2^ = 14.64, p = 0.0001*musculus only(63%)	X^2^ = 11.56, p = 0.002*musculus only(62%)
18646 Da	n.d.^‡^	X^2^ = 1.58, p = 0.40*domesticus* only(13%)	n.d.	X^2^ = 7.80, p = 0.015*domesticus* only(46%)
18651 Da	X^2^ = 53.13, p < 0.0001*domesticus only(97%)	n.d.	X^2^ = 26.66, p < 0.0001*domesticus only(87%)	n.d.
18666 Da	X^2^ = 21.80, p < 0.0001*domesticus only(57%)	z = 0.15, p = 0.91*domesticus* [0.015],*musculus* [0.014]	X^2^ = 15.50, p < 0.0001*domesticus only(60%)	z = 3.51, p = 0.0004*domesticus [0.020]* *>* musculus* [0.004]
18679 Da	X^2^ = 26.92, p < 0.0001*musculus only(63%)	X^2^ = 20.00, p < 0.0001*musculus only(100%)	X^2^ = 18.79, p < 0.0001*musculus only(74%)	X^2^ = 26.00, p < 0.0001*musculus only(100%)
18682 Da	X^2^ = 43.03, p < 0.0001*domesticus only(87%)	X^2^ = 10.00, p = 0.004*domesticus* only(63%)	X^2^ = 26.66, p < 0.0001*domesticus only(87%)	X^2^ = 13.77, p = 0.0002*domesticus only(69%)
18694 Da	z = 2.16, p = 0.031*musculus* [0.28]* *>* domesticus* [0.18]	z = 3.70, p = 0.0002*domesticus [0.56]* *>* musculus* [0.16]	z = 1.96, p = 0.051 *domesticus* [0.12]* *>* musculus* [0.10]	z = 4.33, p < 0.0001*domesticus [0.46]* *>* musculus* [0.13]
18708 Da	z = 6.46, p < 0.0001*domesticus [0.28] > *musculus* [0]	X^2^ = 12.86, p = 0.001*domesticus [0.10]* *>* musculus* [0]	z = 5.01, p < 0.0001*domesticus [0.44]* *>* musculus* [0]	z = 2.67, p = 0.005*domesticus* [0.06]* *>* musculus* [0]
18711 Da	X^2^ = 42.84, p < 0.0001* *musculus* only (85%)	z = 0.61, p = 0.59 *domesticus* [0], *musculus* [0]	X^2^ = 8.26, p = 0.005 *musculus* only (42%)	z = 1.60, p = 0.096 *domesticus* [0.02], *musculus* [0]
18713 Da	n.d.	z = 1.36, p = 0.20*domesticus* [0],*musculus* [0.028]	n.d.	z = 0.95, p = 0.38*domesticus* [0.001],*musculus* [0]
18716 Da	n.d.	X^2^ = 2.35, p = 0.24 *musculus* only (25%)	n.d.	X^2^ = 7.80, p = 0.015 *musculus* only (46%)
18724 Da	X^2^ = 32.25, p < 0.0001*domesticus only(73%)	X^2^ = 20.00, p < 0.0001*domesticus only(100%)	X^2^ = 30.15, p < 0.0001*domesticus only(93%)	X^2^ = 13.77, p = 0.0002*domesticus only(69%)
18731 Da	X^2^ = 57.00, p < 0.0001*musculus only(100%)	z = 3.70, p = 0.0002*musculus [0.18]* *>* domesticus*[0.01]	X^2^ = 34.00, p < 0.0001*musculus only(100%)	z = 4.13, p < 0.0001*musculus [0.29]* *>* domesticus* [0]
18752 Da	X^2^ = 36.94, p < 0.0001*musculus only(78%)	X^2^ = 7.18, p = 0.015*musculus* only(58%)	X^2^ = 34.00, p < 0.0001*musculus only(100%)	X^2^ = 26.00, p < 0.0001*musculus only(100%)

^†^z values from Mann-Whitney U tests examine subspecies differences in the relative amount of each peak, expressed as a proportion of all central MUPs expressed in each sample (median values in square brackets, full data shown in Fig. 6), for mass peaks that were observed in samples from both sub-species. Contingency chi-squared test the frequency of expression (presence/absence) where a mass peak was observed in samples from one subspecies but not in the other subspecies. Presence was recorded only for peaks that were 5% or more of the highest peak in the profile to ensure this reflected real expression rather than baseline noise (% samples meeting this criterion shown in parentheses).

^‡^n.d. = not detected in samples from either subspecies. *P values that would still be statistically significant after sequential Bonferroni adjustment.

**Table 2:** Tests of greater difference between the subspecies in contact versus allopatric samples for the relative intensity of each mass peak.

	Females	Males	Both sexes combined	Candidate (A, R)^††^
(a) *musculus* only expression^‡^				
18575 Da	z = 2.22, p = 0.013	z = 2.55, p = 0.005	z = 3.07, p = 0.001*	A, R
18679 Da	z = −4.75, p > 0.9999	z = −4.74, p > 0.9999	z = −6.60, p > 0.9999	A
18752 Da	z = 1.55, p = 0.06	z = −0.74, p = 0.62	z = −0.46, p = 0.67	A
(b) *domesticus* only expression^‡^				
18651 Da	X^2^ = 32.65, p < 0.0001*	X^2^ = 21.03, p < 0.0001*	X^2^ = 53.90, p < 0.0001*	R
18682 Da	z = 1.93, p = 0.027	z = −0.58, p = 0.71	z = 0.57, p = 0.29	A
18724 Da	z = −4.05, p > 0.9999	z = −1.64, p = 0.95	z = −3.74, p > 0.9999	A
(c) masses expressed by both subspecies^‡‡^				
18666 Da	z = 6.14, p < 0.0001*	z = 3.31, p = 0.0005*	z = 6.81, p < 0.0001*	A, R
18694 Da	z = −3.96, p > 0.9999	z = −1.13, p = 0.87	z = −3.35, p = 0.9996	—
18708 Da	z = 4.44, p < 0.0001*	z = 2.20, p = 0.014	z = 4.87, p < 0.0001*	A, R
18711 Da	z = 5.27, p < 0.0001*	z = 3.72, p = 0.0001*	z = 6.41, p < 0.0001*	R
18731 Da	z = 6.64, p < 0.0001*	z = 4.15, p < 0.0001*	z = 7.80, p < 0.0001*	A, R

Frequencies of expression for each subspecies, sex and origin are given in Supplementary Table S1. Divergence between subspecies is examined in Table 1 for each sex and origin (contact/allopatric).

^‡^For masses expressed by only one subspecies (a, b), the directional prediction of stronger expression in contact versus allopatric samples is assessed by Mann-Whitney U tests, or by contingency chi-squared (when a mass peak was observed in only contact or allopatric samples).

^‡‡^For masses expressed by both subspecies (c), the directional prediction of stronger subspecies divergence in contact versus allopatric populations was tested using this specific a priori contrast for the interaction between subspecies and origin in a 2 × 2 factorial non-parametric ANOVA.

^††^A: assortment: subspecies differences evident both in allopatry and contact; R: reinforcement: subspecies differences more marked in contact than in allopatry.

*Significant after a sequential Bonferroni correction.
