# Supplementary material for: Molecular heterogeneity in major urinary proteins of Mus musculus subspecies: potential candidates involved in speciation
Source: Sci Rep. 2017 Mar 24;7:44992. doi: 10.1038/srep44992 (PMC5364487; doi:10.1038/srep44992)
Supplement: Supplementary Information [file srep44992-s1.pdf]

Supplementary Figures and Table

Molecular heterogeneity in major urinary proteins of *Mus musculus* subspecies: potential candidates involved in speciation

Jane L Hurst, Robert J Beynon, Stuart D Armstrong, Amanda J Davidson, Sarah A Roberts, Guadalupe Gómez-Baena, Carole M Smadja, Guila Ganem

Supplementary Figure S1

Evidence for darcin in *Mus musculus musculus*

Supplementary Figure S2

Detailed peptide mass fingerprinting analysis of Mups from *Mus musculus domesticus*

Supplementary Figure S3

Detailed peptide mass fingerprinting analysis of Mups from *Mus musculus musculus*

Supplementary Table S1

Frequencies of occurrence of each mass peak

Protein identification by peptide mass fingerprinting

Protein bands separable by either SDS-PAGE or by native gel electrophoresis were characterised further by peptide mass fingerprinting, analysing peptides that were the result of cleavage after lysine and arginine residues (trypsin) or just lysine residues (endopeptidase LysC). The masses of many of the derived peptides matched cognate peptides from C57BL/6J MUPs, and we used the C57BL/6J protein sequences as a basis for our analysis. The masses of a few tryptic (prefixed T) or endopeptidase LysC (prefixed L) peptides differed from those obtained by digestion of the known C57BL/6J isoforms. Our analysis explored whether each peptide mass change could be elicited, most simply, by a single amino acid substitution that had been observed previously in known MUPs. Secondly, we assessed whether the combination of mass shift in the differing peptide(s) could explain, in full, the measured intact mass of an observed protein and thus, by inference, the sequence of the protein.

Darcin

Almost all male samples from both subspecies (contact and allopatric populations) exhibited a high mobility band on SDS-PAGE that was absent from female samples (Fig. 2d). This band recapitulated the electrophoretic behaviour of the male peripheral MUP pheromone, darcin[52]. In-gel digestion of the high mobility SDS-PAGE band in *domesticus* and *musculus* confirmed that all the peptides matched the peripheral MUP darcin, and identifies the high mobility band in all male samples as darcin (Supplementary Fig. S1). The 4 Da shift in mass observed in male *domesticus* contact samples (Figure 3) suggests minor amino acid change(s). However, this could not be detected by peptide mass fingerprinting (Supplementary Fig. S1), suggesting that the change(s) occurred in peptides that were too small to measure.

Figure S1

Evidence that the high mobility MUP bands in samples of *M musculus musculus (m….)* and *M musculus domesticus (d….)* are darcin.

Representative peptide mass fingerprint for darcin (red boxed bands) were obtained by in-gel digestion and MALDI-TOF mass spectrometry.

A mass spectrum and associated peptide map is shown as an example on page 2, and an additional 7 peptide maps are presented on page 3. In all instances, coverage was high and confirmed that this band is darcin.

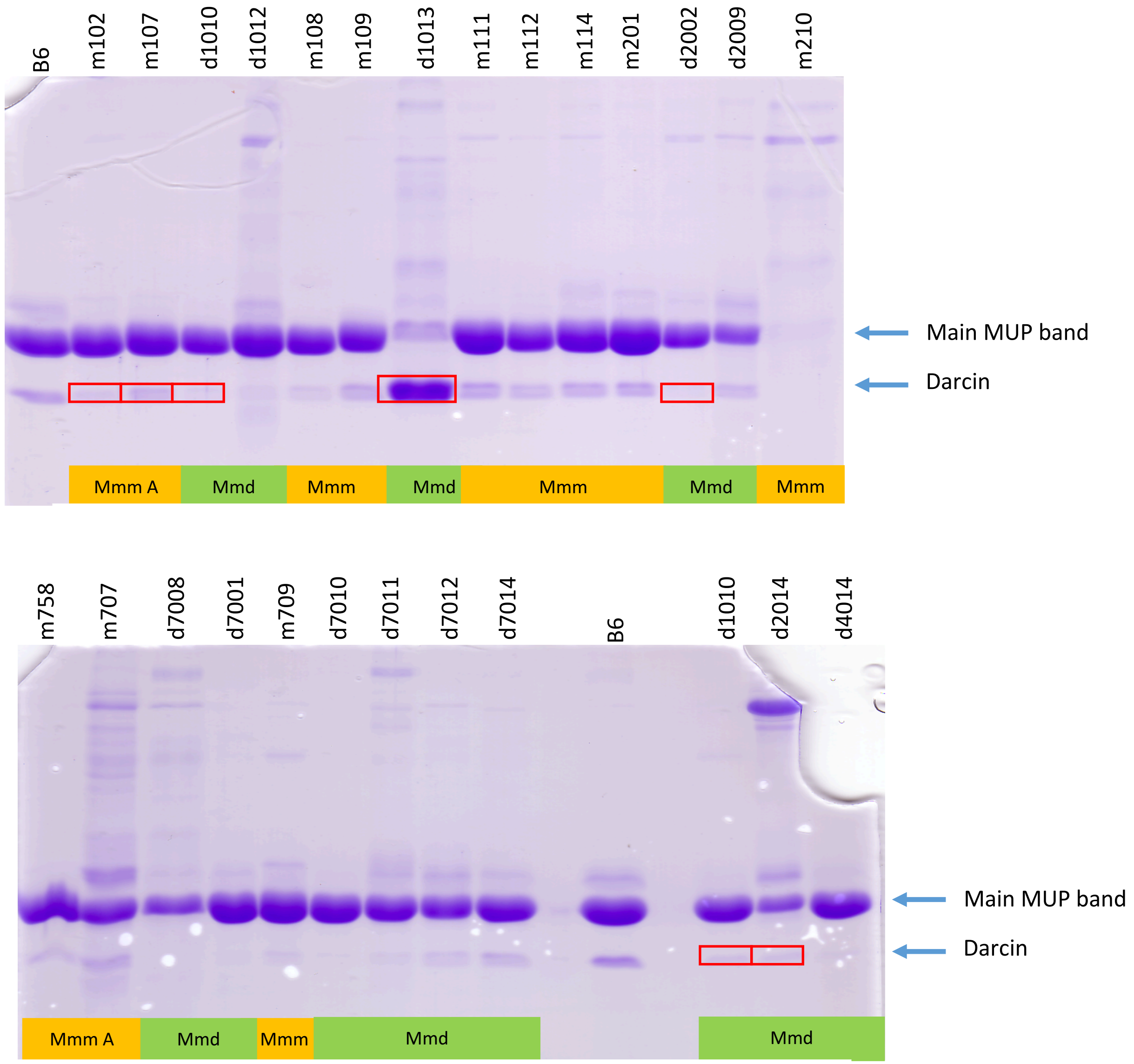

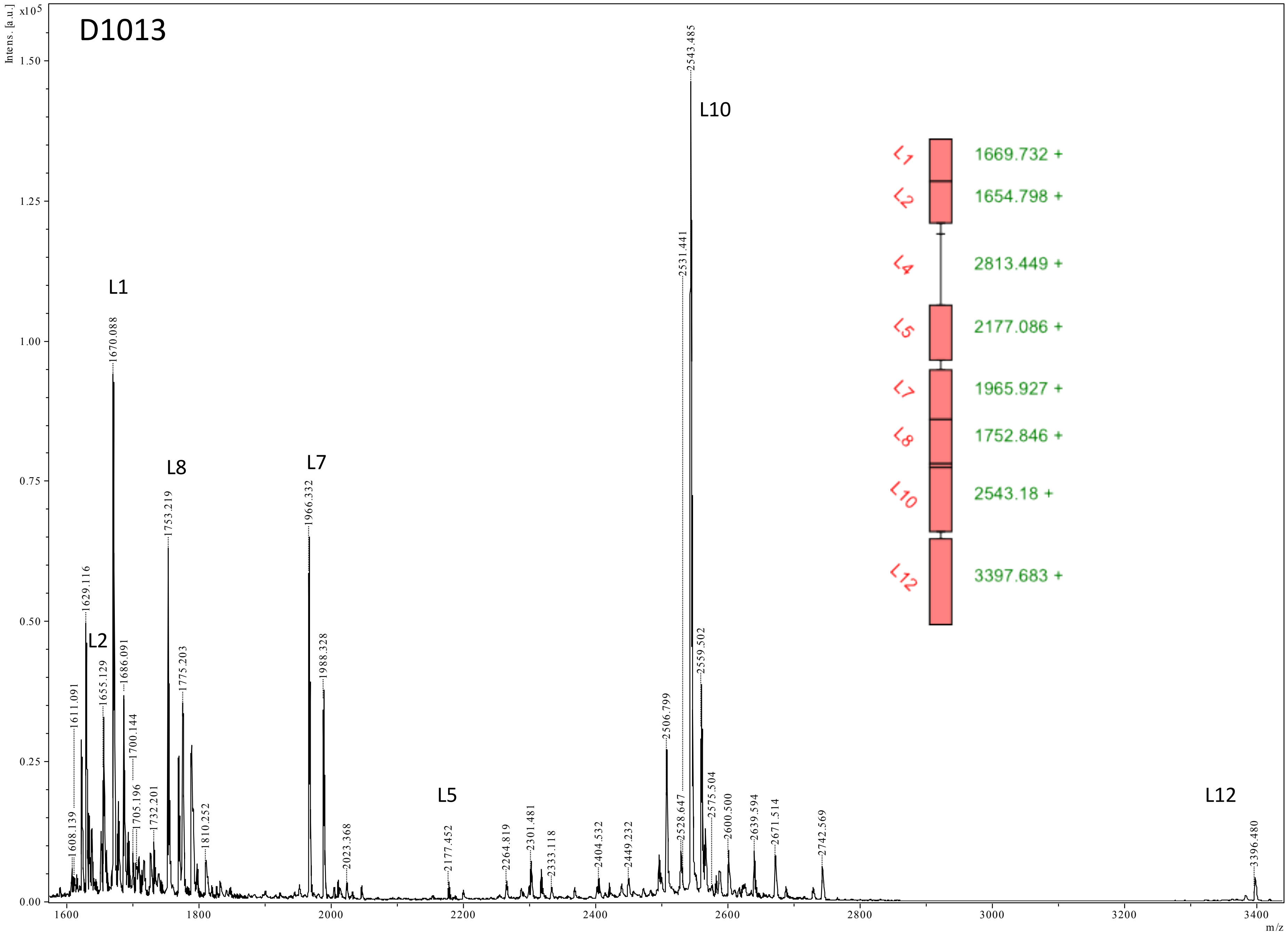

Representative peptide mass fingerprint for darcin (red boxed band cut from gel for *domesticus* mouse D1013 on the previous page.

The peptide map defines the theoretical peptides expected for B6 darcin (MGI_MUP20), if the peptides are observable in each spectrum, they are represented by filled boxes. In this spectrum, L4 was not observed (both L4 and L12 are weakly ionising).

Representative peptide maps that define the theoretical peptides expected for B6 darcin (MGI_MUP20), if the peptides are observable in each spectrum, they are represented by filled boxes. Data are shown for multiple high mobility bands from *musculus* and *domesticus.*

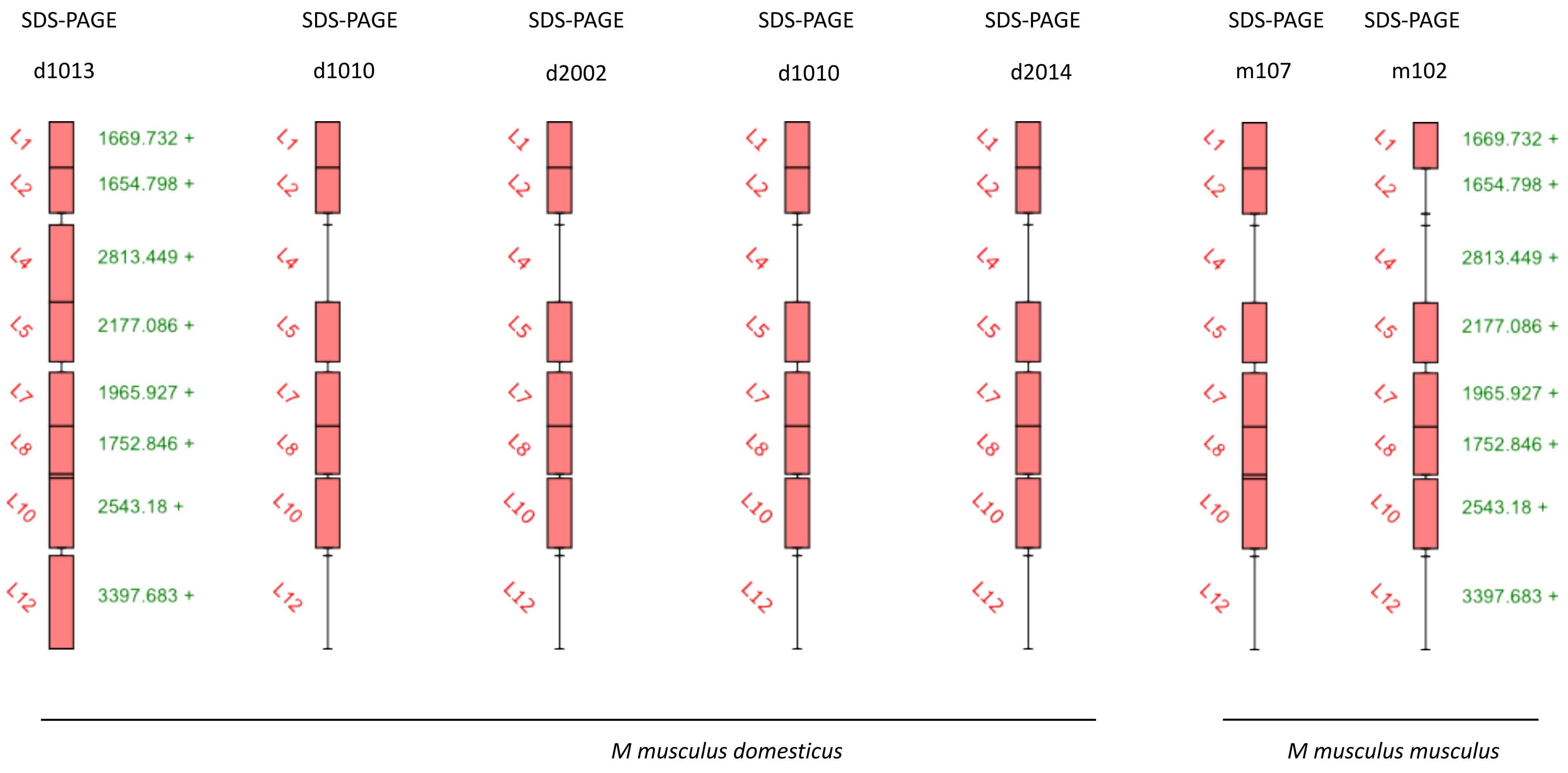

M. m. domesticus central MUPs

Three native gel bands were resolved in both male and female contact *domesticus* (labelled A-C, Supplementary Fig. S2). Each was digested with trypsin or endopeptidase LysC, and the masses of the resultant peptides were determined by MALDI-TOF mass spectrometry. The combination of the two proteases led to almost complete coverage (>90%) of the MUP sequences, apart from small peptides that fell below the lower mass limit of the instrument. Some endopeptidase LysC peptides were larger than 2,500 Da, making them difficult to measure routinely by this technique and, where possible, we used the tryptic digestion to obtain data for these regions of the primary sequence; trypsin cleaves these proteins more frequently and generates smaller fragments. The peptides generated from each band showed a very high degree of matching to those from known MUPs encoded by central *Mup* genes, which differ from each other in only a small number of amino acids. Gel bands of the equivalent mobility from female samples were digested with endopeptidase LysC to confirm correspondence to the equivalent male bands.

For *domesticus* male band A, the peptide mass fingerprints were compared to the protein sequence for MUP7 (18645 Da) as a close match (Supplementary Fig. S2). Two peptides (L5 and T8) differed in mass from the corresponding peptides in MUP7 by +48 Da (L5: m/z 2153 – m/z 2105 = 48 Da; T8: m/z 659 – m/z 611 = 48 Da). This mass shift is completely consonant with a frequently seen amino acid substitution $V_{56}F$ (mature protein numbering[49]. An additional peptide T6 was mass shifted by m/z 1001 – m/z 964 = 37 Da. This mass shift is not explained by a single amino acid substitution, but is consistent with a double amino acid substitution ($D_{34}E$ and $N_{35}H$) seen in MUP17 and another sequence identified from wild *domesticus*[53]. These individual mass shifts (+48, +37) would independently yield MUPs of mass 18645 + 48 = 18693 Da (a sequence corresponding to MUP1 and MUP12) and 18645 + 37 = 18682 Da (a new sequence but involving only previously observed substitutions). Both of these predicted MUP masses were observed in the corresponding intact mass profiles (Fig. 4) and could therefore be derived by these known mass changes. By contrast, the combination of both changes in a single protein sequence would

lead to a predicted mass of 18731 Da, which was not observed in the intact mass profiles of *domesticus* from contact populations.

For band B, the combined peptide mass fingerprints aligned strongly with MUP10 (18708 Da) and no mass shifted peptides were evident (Supplementary Fig. S2). From this, we conclude that band B contained the protein responsible for the observed 18708 Da intact mass peak, and is very likely to be MUP10.

Male band C yielded peptide mass fingerprints that largely matched the commonly observed MUP at 18694 Da, encoded by at least five genes in the C57BL/6J mouse (*Mup9, Mup11, Mup16, Mup18, Mup19*). Two mass-shifted peptides were apparent; L5/T8 was consistent with the common $F_{56}V$ substitution (see above) and T6 had the same +37 Da mass shift observed for band A (consistent with $D_{34}N_{35}$ to $E_{34}H_{35}$). Individually, these mass shifts would yield proteins at 18694 – 48 = 18646 Da and 18694 + 37 = 18731 Da, but neither of these were observed in the intact mass analysis. However, the combination of both mass shifts gives a predicted protein mass of 18694 – 48 + 37 = 18683 Da, which corresponds closely to the observed mass at 18682 Da (Fig. 4). In addition, the L4 peptide observed is evidence of $D_{34}N_{35}$ and thus, can only be explained by other MUP(s) in this band that possess this particular dipeptidyl sequence.

Three electrophoretic bands were also observed in female *domesticus*, (also labelled A-C, Supplementary Fig. S2), and yielded endopeptidase LysC peptide mass fingerprints that were fully consistent with the matching male bands. Overall, these data confirmed the likely amino acid changes that would lead to five different MUP isoforms with three observed protein masses, at 18708 Da (band B), at 18682/3 Da (bands A and C) and at 18693/4 Da (bands A and C).

Supplementary Figure S2 | Peptide mass fingerprinting of MUPs from *Mus musculus domesticus*

MUPs were resolved by native gel electrophoresis from representative male and female urine samples from contact *domesticus* mice (top right, urine from a male C57BL/6J (B6) mouse is also included for reference). The averaged intact mass profiles for contact *domesticus* samples are shown to the right of the gels. From these gels, specific bands (labelled A - C) were subjected to in-gel digestion with trypsin or endopeptidase LysC followed by peptide mass fingerprinting by MALDI-TOF mass spectrometry. From the resultant peptide masses, peptide maps were reconstructed by reference to representative MUPs in the C57BL/6J genome of known sequence, to identify mass shifted peptides. The theoretical peptide map for each reference MUP is presented and observed peptide masses are shaded in pink (trypsin) and light blue (endopeptidase LysC). Where peptides of different masses were observed (highlighted as boxes below the predicted peptide mass: dark red (trypsin), dark blue (endopeptidase LysC)), they were used to predict the average mass of the intact protein, for comparison with the intact mass profiles. Where multiple mass shifted peptides were observed, they could also be combined to generate a new predicted protein mass (orange boxes). For new predicted mass, those that were observed are boxed, whilst the predicted masses that were not observed are indicated in grey text.

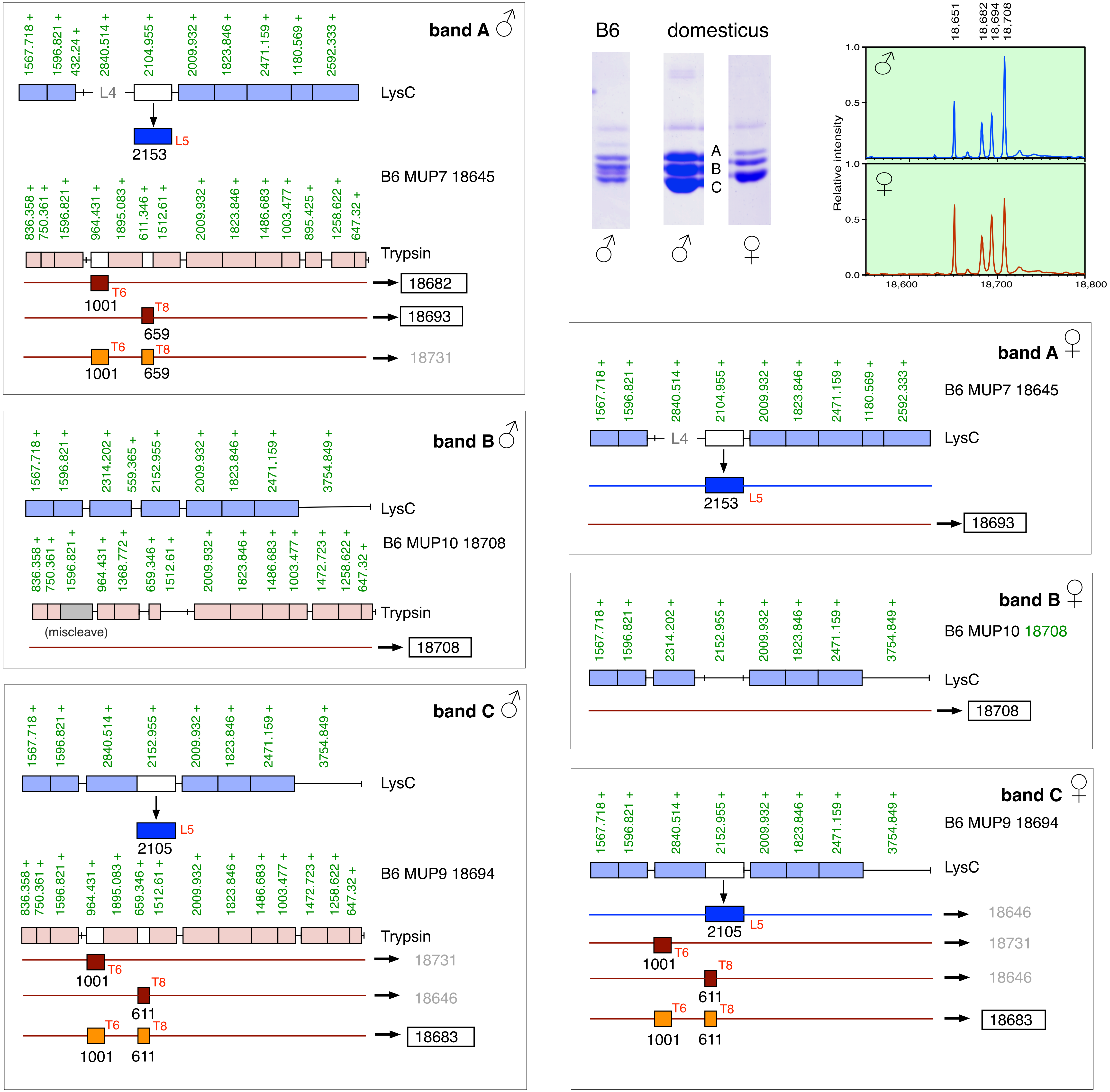
band A ♂
LysC
L4
L5
2153
B6 MUP7 18645
Trypsin
T6
T8
1001
659
18682
18693
18731
B6
domesticus
A
B
C
♂
♀
18,651
18,682
18,694
18,708
Relative intensity
18,600
18,700
18,800
band B ♂
B6 MUP10 18708
(miscleave)
18708
band C ♂
2105
B6 MUP9 18694
611
18646
18683
band A ♀
band B ♀
band C ♀

M. m. musculus central MUPs

Urinary proteins of male and female *musculus* resolved into two major electrophoretic bands by native gel electrophoresis (Supplementary Fig. S3), one much more intensely stained than the other. The peptide mass fingerprint from male *musculus* band D was mapped against MUP7 (18645 Da) but revealed a series of mass shifted peptides. Peptides L4 and T6 revealed the same +37 Da mass shift observed in *domesticus* above (most likely $D_{34}N_{35}$ changed to $E_{34}H_{35}$). Peptides T8, T9 and L5 revealed two mass shifted amino acid substitutions. The first is consistent with the $V_{56}F$ substitution (+48 Da) commonly observed in *domesticus* MUPs. The second, in T9, was a 22 Da mass shift that would be consistent with an Asp to His amino acid change. This substitution has not previously been reported in any *domesticus* MUP sequence (nor any other variation in the T9 peptide). The combination of these two mass shifts (48 + 22 = 70 Da) was identical to the difference in L5 between the *musculus* MUP and MUP7 from C57BL/6J (2175 - 2105 = 70 Da). These amino acid changes combined would yield a protein of predicted mass 18752 Da, exactly consonant with the intact mass peak of 18752Da observed in *musculus,* predominantly expressed by males (Supplementary Fig. S3).

M. m. musculus band E yielded comprehensive LysC and trypsin peptide maps that mapped well to the 18694 Da MUP that is encoded by five genes in C57BL/6 (*Mup9, Mup11, Mup16, Mup18, Mup19*), and there was a corresponding peak at 18694 Da in intact mass profiles. In addition, the peptide fingerprint revealed a single mass shifted peptide (T6), comparable to the previously observed +37 Da mass shift seen with *domesticus* samples (most likely $D_{34}N_{35}$ changed to $E_{34}H_{35}$). The protein mass predicted from this amino acid change is 18731 Da, exactly consonant with the main protein peak evident in male and female *musculus* intact mass profiles (Supplementary Fig. S3).

Supplementary Figure S3 | Peptide mass fingerprinting of MUPs from *Mus musculus musculus*

MUPs were resolved by native gel electrophoresis from representative male and female urine samples from contact *musculus* mice (top right, urine from a male C57BL/6J mouse is also included for reference). The averaged intact mass profiles for contact *musculus* samples are shown to the right of the gels. From these gels, specific bands (labelled D, E) were subjected to in-gel digestion with trypsin or endopeptidase LysC followed by peptide mass fingerprinting by MALDI-TOF mass spectrometry. From the resultant peptide masses, peptide maps were reconstructed by reference to representative MUPs in the C57BL/6J genome of known sequence, to identify mass shifted peptides. The theoretical peptide map for each reference MUP is presented and observed peptide masses are shaded in pink (trypsin) or light blue (endopeptidase LysC). Where peptides of different masses were observed (highlighted as boxes below the predicted peptide mass: dark red (trypsin), dark blue (endopeptidase LysC)), they were used to predict the average mass of the intact protein, for comparison with intact mass profiles. Where multiple mass shifted peptides were observed, they could also be combined to generate a new predicted protein mass (orange boxes). For new predicted mass, those that were observed are boxed, whilst the predicted masses that were not observed are indicated in grey text.

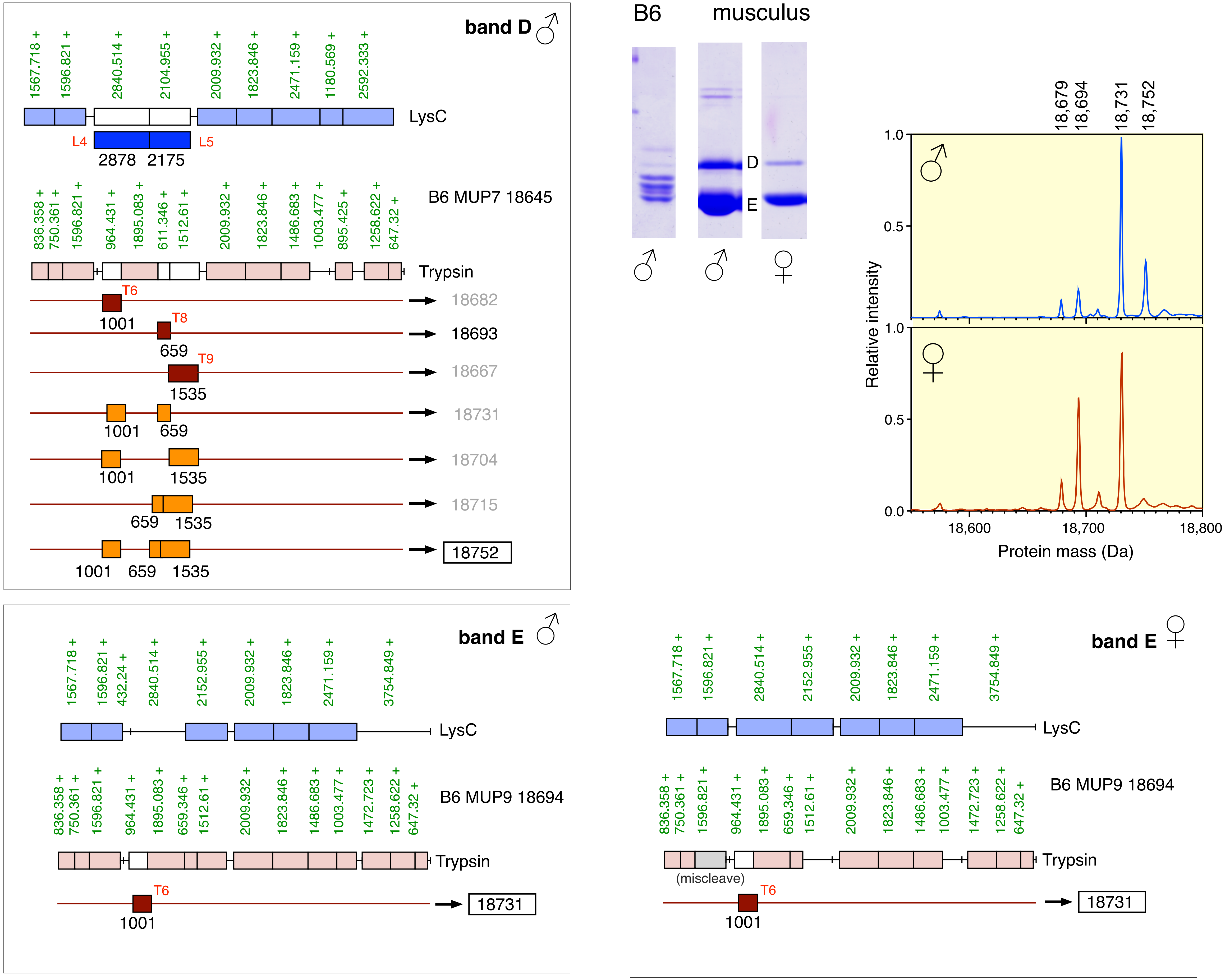

band D ♂
1567.718 + 1596.821 + 2840.514 + 2104.955 + 2009.932 + 1823.846 + 2471.159 + 1180.569 + 2592.333 +
LysC
L4 L5
2878 2175
B6 MUP7 18645
836.358 + 750.361 + 1596.821 + 964.431 + 1895.083 + 611.346 + 1512.61 + 2009.932 + 1823.846 + 1486.683 + 1003.477 + 895.425 + 1258.622 + 647.32 +
Trypsin
T6 1001 18682
T8 659 18693
T9 1535 18667
1001 659 18731
1001 1535 18704
659 1535 18715
1001 659 1535 18752
B6
musculus
D
E
♂ ♂ ♀
18,679 18,694 18,731 18,752
Relative intensity
1.0
0.5
0.0
18,600 18,700 18,800
Protein mass (Da)
band E ♂
1567.718 + 1596.821 + 432.24 + 2840.514 + 2152.955 + 2009.932 + 1823.846 + 2471.159 + 3754.849 +
LysC
B6 MUP9 18694
836.358 + 750.361 + 1596.821 + 964.431 + 1895.083 + 659.346 + 1512.61 + 2009.932 + 1823.846 + 1486.683 + 1003.477 + 1472.723 + 1258.622 + 647.32 +
Trypsin
T6 1001 18731
band E ♀
1567.718 + 1596.821 + 2840.514 + 2152.955 + 2009.932 + 1823.846 + 2471.159 + 3754.849 +
LysC
B6 MUP9 18694
836.358 + 750.361 + 1596.821 + 964.431 + 1895.083 + 659.346 + 1512.61 + 2009.932 + 1823.846 + 1486.683 + 1003.477 + 1472.723 + 1258.622 + 647.32 +
Trypsin
(miscleave)
T6 1001 18731

Table S1: Frequencies of occurrence of each mass peak (at least 5% of highest peak in the intact mass profile)

Mass (Da)	females				males			
	contact		allopatric		contact		allopatric	
	musculus	*domesticus*	*musculus*	*domesticus*	*musculus*	*domesticus*	*musculus*	*domesticus*
18575 Da	14/27<br>52%	0/30<br>0%	4/12<br>33%	0/8<br>0%	12/19<br>63%	0/15<br>0%	8/13<br>62%	0/13<br>0%
18646 Da	0/27<br>0%	0/30<br>0%	0/12<br>0%	1/8<br>13%	0/19<br>0%	0/15<br>0%	0/13<br>0%	6/13<br>46%
18651 Da	0/27<br>0%	29/30<br>97%	0/12<br>0%	0/8<br>0%	0/19<br>0%	13/15<br>87%	0/13<br>0%	0/13<br>0%
18666 Da	0/27<br>0%	17/30<br>57%	3/12<br>25%	2/8<br>25%	0/19<br>0%	9/15<br>60%	1/13<br>8%	7/13<br>54%
18679 Da	17/27<br>63%	0/30<br>0%	12/12<br>100%	0/8<br>0%	14/19<br>74%	0/15<br>0%	13/13<br>100%	0/13<br>0%
18682 Da	0/27<br>0%	26/30<br>87%	0/12<br>0%	5/8<br>63%	0/19<br>0%	13/15<br>87%	0/13<br>0%	9/13<br>69%
18694 Da	27/27<br>100%	30/30<br>100%	12/12<br>100%	8/8<br>100%	17/19<br>89%	14/15<br>93%	13/13<br>100%	13/13<br>100%
18708 Da	3/27<br>11%	29/30<br>97%	0/12<br>0%	6/8<br>75%	8/19<br>42%	15/15<br>100%	1/13<br>8%	7/13<br>54%
18711 Da	23/27<br>85%	0/30<br>0%	3/12<br>25%	1/8<br>13%	8/19<br>42%	0/15<br>0%	1/13<br>8%	4/13<br>31%
18713 Da	0/27<br>0%	0/30<br>0%	6/12<br>50%	1/8<br>13%	0/19<br>0%	0/15<br>0%	5/13<br>38%	2/13<br>15%
18716 Da	0/27<br>0%	0/30<br>0%	3/12<br>25%	0/8<br>0%	0/19<br>0%	0/15<br>0%	6/13<br>46%	0/13<br>0%
18724 Da	0/27<br>0%	22/30<br>73%	0/12<br>0%	8/8<br>100%	0/19<br>0%	14/15<br>93%	0/13<br>0%	9/13<br>69%
18731 Da	27/27<br>100%	0/30<br>0%	12/12<br>100%	2/8<br>25%	19/19<br>100%	0/15<br>0%	13/13<br>100%	5/13<br>38%
18752 Da	21/27<br>78%	0/30<br>0%	7/12<br>58%	0/8<br>0%	19/19<br>100%	0/15<br>0%	13/13<br>100%	0/13<br>0%
